# Influence of Polypropylene Fibres on Energy Dissipation Mechanisms and Thermo-Chemical Degradation of Cement Mortars Subjected to High Temperatures

**DOI:** 10.3390/ma19163440

**Published:** 2026-08-13

**Authors:** Tomasz Drzymała, Bartosz Zegardło, Sylwia Lewicka, Krzysztof Przystupa, Ewa Rudnik

**Affiliations:** 1 Faculty of Safety Engineering and Civil Protection, Fire University, 52/54 Słowackiego Street, 01-629 Warsaw, Poland; 2 Institute of Computer Science, Faculty of Natural Sciences, University of Siedlce, 3 Maja 54 Street, 08-110 Siedlce, Poland; bartosz.zegardlo@uws.edu.pl; 3 Independent Researcher, 41-803 Zabrze, Poland; lewicka.sylwia137@gmail.com; 4 Department of Automation, Lublin University of Technology, Nadbystrzycka 38D, 20-618 Lublin, Poland; 5 Institute of Safety Engineering, Fire University, 52/54 Słowackiego Street, 01-629 Warsaw, Poland; erudnik@apoz.edu.pl

**Keywords:** safety engineering, cementitious composite, cement mortars, polypropylene fibres, effect of high temperature, mechanical properties, microstructure and mechanical properties, thermal spalling

## Abstract

This article is a continuation of research conducted by the authors on the effects of fire on cementitious composites and presents findings of an investigation into cement mortars that incorporate monofilament (I) and multifilament (F) polypropylene fibres following exposure to temperatures between 100 and 600 °C. Research was undertaken to examine the effect of adding fibre on the mechanical performance, microstructural characteristics, and thermochemical degradation behaviour of the mortars under conditions representative of high-temperature exposure during fires in energy infrastructure facilities. The scope of the research comprises establishing the modulus of elasticity using dog-bone-shaped specimens, as well as flexural and compressive strength tests performed on prisms measuring 4 × 4 × 16 cm and on 10 × 10 × 10 cm cubes to determine the strength class of the mortars. Microstructural analyses complemented the mechanical testing, performed with the use of scanning electron microscopy (SEM); this made it possible to assess temperature-induced changes in the cement matrix. The results have demonstrated that polypropylene fibres had a significant influence on the degradation behaviour of mortars subjected to elevated temperatures, particularly those between 200 and 400 °C, where fibre melting promoted the formation of additional pore channels. This phenomenon promotes the dissipation of internal energy associated with boiling water vapour contained in the capillary pores, as well as water released during the dehydration of cement hydration products, thereby limiting rapid pressure build-up and reducing the risk of explosive spalling. Moreover, the observed microstructural changes were associated with progressive decomposition of C–S–H gels and other thermo-chemical processes occurring within the cement matrix. The results confirm that polypropylene fibres act as a passive mechanism for the dissipation of thermal and mechanical energy in cement mortars, which has a positive effect on their performance under high-temperature conditions. The study provides new experimental data of significance for the design of cement-based materials with enhanced resistance to thermal exposure in energy-sector facilities.

## 1. Introduction

Energy infrastructure facilities, such as power plants, industrial facilities, utility tunnels and energy storage installations, are particularly vulnerable to the effects of elevated temperatures in emergency conditions, including fire events. In such scenarios, structural materials are exposed to significant thermal energy fluxes, which lead to rapid changes in their mechanical properties and durability. Cement-based composites, commonly used in such facilities, exhibit a complex response to thermal energy exposure stemming from both physical and chemical processes occurring within their microstructure. Increasing temperature promotes rapid evaporation of moisture retained within the pore structure of the material, while simultaneously causing a gradual dehydration of cement hydration products. As a result, water vapour accumulates within the pores, leading to a rise in internal pore pressure. What is more, exposure to elevated temperatures triggers chemical changes in the cement matrix, including the decomposition of C–S–H gels and other binding phases that are essential for cohesion of the material and load-bearing capacity. Collectively, these thermochemical transformations cause progressive degradation of the internal structure of cementitious composites, resulting in reduced strength and stiffness. During rapid heating, the accumulated energy of water vapour may become released in an uncontrolled manner, causing localised damage, surface delamination, and explosive spalling. These phenomena are a significant threat to the integrity of structural elements, particularly in facilities where operational continuity and the safety of energy installations are critical. Understanding the interplay between thermal energy exposure, chemical transformations occurring within the cement matrix, and the mechanical response of the material is therefore a key issue in the design and durability assessment of cement-based composites used in energy infrastructure.

The processes occurring in concrete and other cement-based composites under elevated temperature conditions have been the subject of extensive scientific research for a number of years [1,2,3,4,5,6,7,8]. Interest in this topic is primarily focused on two related, yet distinct, research areas. The first of these concerns cement-based materials designed for long-term service under sustained high-temperature exposure, as is the case, among others, for industrial chimney linings or components of technological installations [9]. The second area involves an analysis of the behaviour of cement composites in situations where exposure to high temperature is incidental and accidental, which is typical of fire scenarios [10].

In industrial practice, particularly in the technology of refractory ready-mix and specialty concretes, numerous composites are used that meet the requirements for long-term service under extremely high-temperature conditions. By using carefully selected additives and chemical admixtures, cementitious materials can be formulated to withstand long-term service at temperatures of up to 1500 °C, whilst maintaining their safe performance characteristics. Nevertheless, such composites need to be exposed to elevated temperatures progressively, allowing the physical and chemical changes within the cement matrix to develop in a controlled and predictable manner.

When such cement-based composites are exposed to elevated temperatures, cement hydration products undergo a sequence of dehydration reactions, particularly those involving C–S–H gel, portlandite (Ca(OH)_2_), and hydrated calcium aluminates and aluminosilicates. At temperatures up to approximately 200 °C, these transformations occur mainly as a result of a release of physically bound water, which may be described by the following general reaction:

C–S–H⋅nH_2_O → C–S–H + nH_2_O↑ (1)

A continued increase in temperature promotes the gradual breakdown of hydration bonds and the consequent deterioration of the C–S–H gel structure. This process leads to a significant change in the nature of interfacial interactions. Chemically and hydration-induced bonds become progressively replaced by ionic–covalent bonds characteristic of ceramic phases.

Within the temperature range of approximately 400–550 °C, dehydroxylation of portlandite occurs:

Ca(OH)_2_ → CaO + H_2_O↑ (2)

This process is accompanied by an increase in porosity and local weakening of the cement matrix structure. Simultaneously, further reorganisation of the silicate structure takes place, which leads to the formation of more stable anhydrous silicate phases. In the presence of mineral additives rich in SiO_2_ and Al_2_O_3_, secondary reactions may also occur, such as:

CaO + SiO_2_ → CaSiO_3_ (wollastonite) (3)

2CaO + Al_2_O_3_ + SiO_2_ → Ca_2_Al_2_SiO_7_ (gehlenite) (4)

CaO + Al_2_O_3_ + 2SiO_2_ → CaAl_2_Si_2_O_8_ (anorthite) (5)

These processes are similar in nature to ceramic sintering, in which diffusion mechanisms and crystal lattice reorganisation play a dominant role, leading to the formation of stable high-temperature phases with enhanced chemical and thermal stability. Up to 160 h may be required for the full sequence of these transformations to be completed, with their completion being essential for ensuring a long-term chemical and structural stability of the material at elevated temperatures [11,12,13]. By contrast, in fire conditions, although cementitious composites are exposed to comparatively lower temperatures, the extremely rapid temperature increase prevents thermochemical reactions from reaching equilibrium. Consequently, the development of materials that offer complete resistance to such thermal stresses remains a significant challenge.

The response of composites, particularly cement-based materials, to elevated temperatures is governed by complex physicochemical phenomena associated with their multiphase nature and the different levels of thermal stability exhibited by their individual constituents [14,15]. Studies investigating fire exposure have demonstrated that cementitious composites experience modifications to both their external and internal structure, the extent of which is determined primarily by the peak temperature reached and the duration of thermal exposure [16,17,18,19]. These changes are accompanied by substantial alterations in the engineering and performance characteristics of the material [20].

Mechanical properties of coarse-grained concrete at elevated temperatures depend on changes occurring simultaneously in the cement paste, aggregate, and the interfacial transition zone (ITZ) between aggregate and paste under thermal exposure [21,22,23]. During the heating of cement composites to temperatures of approximately 400 °C, processes taking place within the cement matrix play a dominant role [24,25,26]. The initial stage of heating, up to approximately 100 °C, is characterised first and foremost by the evaporation of free water. At ca. 180 °C, physically bound water associated with cement hydration products becomes progressively eliminated. Further temperature increase to approximately 300–400 °C causes a release of chemically bound water due to the thermal decomposition of portlandite, one of the principal crystalline phases produced during cement hydration.

In parallel, the cement matrix, containing hydration products of varying thermal stability, undergoes significant microstructural transformations. In particular, depolymerisation processes of calcium silicate hydrate (C–S–H) gels are initiated, which are responsible for the load-bearing capacity and cohesion of the cement matrix. This process involves a reduction in the degree of polymerisation of silicate chains, their shortening, and a decrease in the Ca/Si ratio resulting from the progressive decalcification of the C–S–H phases:

Ca_x_Si_y_O_z_⋅H_2_O (C–S–H) → Ca_x−n_Si_y_O_z_⋅H_2_O(C–S–H with a lower Ca/Si) + nCa^2+^ (6)

C–S–H → C–S–H _lower Ca/Si_ + Ca ^2+^ (7)

At higher temperatures, the amorphous or poorly crystalline structure of C–S–H becomes partially transformed into more thermodynamically stable calcium silicate phases, accompanied by matrix volumetric shrinkage and the initiation of microcracks. At temperatures over ca. 400 °C, processes occurring in the aggregate become increasingly significant. In concretes containing siliceous aggregate, an allotropic transformation of quartz from the low-temperature α form to the high-temperature β form takes place at ca. 573 °C:

α-SiO_2_ → β-SiO_2_ (8)

This transformation is associated with an abrupt increase in the volume of aggregate particles, which leads to the development of significant internal stresses and degradation of the bond at the aggregate–cement paste interface.

Portland cement concretes are generally considered to retain most of their mechanical performance at temperatures of up to ca. 300 °C. Above this level, the material undergoes progressive structural deterioration, resulting in a loss of strength and the development of permanent deformation. In general, complete structural failure does not take place until temperatures reach approximately 500–600 °C or higher [16]. The deterioration in the mechanical performance of concrete exposed to elevated temperatures is primarily attributed to the differential thermal deformation of the aggregate and the cement paste, which weakens the bond within the interfacial transition zone. This phenomenon becomes increasingly pronounced in concretes containing coarse aggregate.

The mismatch in volumetric changes is further amplified by chemical reactions occurring simultaneously within the cement paste and aggregate phases. The severity of their influence on concrete behaviour depends largely on the heating rate and the peak temperature attained during thermal exposure. The cooling method also has a significant influence on the degree of strength degradation. Concrete elements subjected to rapid cooling, such as water quenching or immersion, typically exhibit a much greater reduction in strength than elements cooled naturally in air. Some studies have reported a gradual partial recovery in the strength of concrete following heating [27]. This phenomenon has been attributed to the delayed rehydration of free lime (CaO) formed as a result of the thermal decomposition of portlandite, as well as to further reorganisation of the cement matrix microstructure. This process is characterised by the hydration of calcium oxide, which causes the re-formation of portlandite, as represented by the following equation:

CaO + H_2_O → Ca(OH)_2_ (9)

Portlandite formed as a result of rehydration may partially fill pores and microcracks, promoting a local improvement in the structural cohesion of the cement matrix, while simultaneously enabling further reorganisation of decalcified C–S–H phases.

The loss of concrete strength at elevated temperatures has been the subject of extensive research [28,29,30,31,32,33,34,35,36,37,38]. These works have focused both on identifying the phenomena occurring in concrete under elevated temperature conditions and on assessing the load-bearing capacity of concrete elements after thermal exposure. In some of these studies, including the work of K. D. Hertz [39], empirical relationships were proposed to estimate the loss of concrete strength as a function of temperature. Extensive studies have been performed on different concrete classes, including normal-strength concrete (NSC) and high-strength concrete (HSC), which exhibit differences in the phase composition of the cement matrix and its degree of densification [40].

The response of concrete structures at higher temperatures depends to a large extent on the flexural modulus of elasticity, which is highly susceptible to thermal effects. The study presented in [41] examines the influence of temperature exposure and aggregate type on variations in the flexural modulus across different concrete mixtures. The experimental results indicate that concretes containing limestone or basalt aggregates are characterised by greater resistance to high-temperature exposure compared to concretes with aggregates of high silica content. This phenomenon is largely attributed to the chemical and phase characteristics of aggregates, as siliceous aggregates such as quartz sand undergo significant structural transformations already at temperatures of around 350 °C, including softening and partial melting. This promotes the degradation of the aggregate–cement paste interfacial transition zone, resulting in a loss of overall composite stiffness.

One of the key destructive phenomena leading to damage of concrete elements under fire conditions is spalling [42,43,44,45,46]. Spalling is defined as the detachment and shedding of near-surface layers of concrete, caused by a rapid increase in pressure of water and water vapour trapped within the material pores. When exposed to temperature changes, water contained in the capillary pores of concrete evaporates and boils, and the resulting expansion of water vapour within the confined space of the pores causes the development of tensile stresses in the surrounding pore walls. Once these stresses exceed the tensile strength of concrete, irreversible damage develops in the form of microcracking, fissuring, and cracking, potentially leading to sudden spalling of the material. It is widely recognised that the spalling mechanism cannot be attributed merely to water vapour pressure, as it results from the interaction of multiple factors. Its occurrence is additionally influenced by pronounced temperature gradients between the surface and the interior of the element, differences in thermal expansion coefficients between the aggregate and cement paste, incompatibility of thermal strains between concrete and steel reinforcement, as well as mineralogical transformations taking place within the components of the cement composite. Collectively, these phenomena contribute to the accumulation of thermal and mechanical stresses both in the surface layer and throughout the volume of the element. An analysis of the described phenomena indicates that their destructive character intensifies with increasing density and degree of compaction of the cement composite. Despite their high strength under normal conditions, concretes with a dense microstructure and low permeability are particularly susceptible to rapid release of internal water vapour energy, which promotes the initiation and intensification of spalling.

Extensive investigations on coarse-grained normal-strength concrete have shown that the incorporation of polypropylene (PP) fibres can improve the behaviour of concrete elements subjected to elevated temperatures and reduce the severity of spalling phenomena [47,48,49,50]. The effectiveness of this approach has also been demonstrated in studies related to the Channel Tunnel Rail Link and through findings obtained within the UPTUN project [37]. The introduction of PP fibres influences the mechanical properties of concrete under both ambient and elevated temperature conditions [28]. In cement-based composites used in normal conditions, polypropylene fibres act as dispersed reinforcement, limiting the development of microcracks and improving the ductility of the material. In contrast, in elements exposed to high temperatures, their thermal behaviour plays a key role. When temperatures approach the polypropylene melting point, the fibres undergo melting, resulting in the formation of pore channels that create a network of pathways for the transport of water vapour accumulated within the capillary structure of the cement paste. This mechanism facilitates a gradual release of vapour and partial dissipation of internal energy, thereby limiting excessive increases in pore pressure and reducing the likelihood of spalling damage.

The latest findings reported in the literature concerning the degradation of cement-based composites at elevated temperatures indicate substantial differences in material behaviour, depending on the binder type, microstructure, and dispersed reinforcement characteristics [51,52,53,54,55]. This variability results from the differing thermal stability of hydration products and the reactions occurring within the cement matrix as temperature increases.

Lima et al. (2025) [51] investigated the influence of temperatures up to 800 °C on lime–cement mortars, for which use a combination of destructive and non-destructive testing methods have been made, including ultrasonic pulse velocity (UPV) measurements. The findings revealed a pronounced deterioration in mechanical performance above 600 °C, with compressive strength decreasing by approximately 87% and UPV values declining by nearly 75%.

Le et al. (2025) [52] focused their study on alkali-activated slag (AAS) mortars, comparing their behaviour with that of traditional Portland cement (PC) mortars. For AAS mortars, the authors observed a 36.9% increase in compressive strength after exposure to 200 °C, which was attributed to the ongoing hydration reactions and the rearrangement of binding phases. However, when temperatures reached 600–800 °C, a rapid deterioration in both compressive strength and abrasion resistance was recorded, resulting from the degradation of the gel structure. A partial recovery of mechanical properties was observed over 800 °C, at temperatures favourable for crystallisation of akermanite.

The most extensive analysis of the influence of fibre reinforcement on the behaviour of mortars at elevated temperatures was presented by Ezziane et al. (2025) [53]. The study examined mortars produced using three different binder systems: Portland cement CEM I, CEM I modified with 8% silica fume, and blast-furnace slag cement CEM III/A, both without fibre reinforcement and with fibre incorporation. The results demonstrated that the addition of fibres improved compressive strength, flexural strength, and elastic modulus over the entire temperature range considered.

The study by Han et al. (2025) [54] contributed to a better understanding of the behaviour of polyethylene fibre-reinforced ultra-high-performance concrete (UHPC), particularly in terms of its resistance to explosive spalling. A key role was played by phase transformations of polyethylene fibres, which at approximately 155 °C undergo melting, followed by partial degradation and volatilisation. This process led to the formation of additional porosity within the composite structure, reaching approximately 5.89% at 400 °C, which enabled effective dissipation of internal water vapour energy and a reduction in pore pressure. As a result, UHPC maintained high compressive strength values, amounting to 98.3 MPa after heating to 400 °C and 36.0 MPa after exposure to 800 °C, while thermal spalling was completely eliminated even at 1000 °C. The authors developed a quantitative model of thermal damage mechanisms, suggesting that thermal stresses and water vapour pressure account for 43.6% and 56.4% of spalling intensity, respectively [54].

Related aspects were examined by Kong et al. (2025) [55]. Their studies focused on residual properties of UHPC containing hybrid steel–polypropylene fibre reinforcement after rapid heating exposure. The results demonstrate that increasing the steel fibre content significantly improves the strength of UHPC, particularly at temperatures below 400 °C, where the fibres effectively restrain the initiation and propagation of microcracks, thereby preserving the structural integrity of the material. At higher temperatures, however, the beneficial effect of reinforcement gradually diminishes due to partial degradation of the polymer fibres and deterioration of the interfacial transition zone between the fibres and the matrix.

A synthesis of the results presented in studies [51,52,53,54,55] indicates that unreinforced mortars and concretes undergo intensive degradation after exceeding temperatures of approximately 600 °C. In this range, a reduction in compressive strength of 70–90% may be observed, resulting from advanced dehydration processes of hydration products, decomposition of binding phases, and progressive microstructural damage. In contrast, cement-based composites modified with fibres, both steel and polymer (polyethylene and polypropylene), exhibit significantly better structural stability and higher residual modulus of elasticity. Our previous studies [56] have demonstrated the positive effect of polypropylene fibres on the high-temperature behaviour of fine-grained building mortars. With the influence of polypropylene (PP), fibre incorporation influence on the thermal performance of cement mortar exposed to elevated temperatures was investigated with the deployment of simultaneous thermal analysis. The results have shown that the addition of PP fibres improved the thermal stability of the mortar after initial heating at 200 °C and 300 °C. In the subsequent study [57], combined simultaneous thermal analysis (STA) and tensile testing were employed to clarify the role of polypropylene fibres in high-strength, fine-grained mortars exposed to fire conditions. Based on the experimental results, a comprehensive statistical analysis was carried out, which led to the development of detailed temperature–parameter relationships.

This study aims to elucidate the mechanisms responsible for the mechanical and microstructural degradation of polypropylene fibre-modified, high-strength fine-grained cement mortars exposed to simulated elevated-temperature conditions. Mechanical properties tests were carried out, including tensile, flexural and compressive tests combined with detailed statistical analysis and microscopic observations using scanning electron microscopy (SEM). Various temperatures ranging from 100 °C to 600 °C were applied. Based on the authors’ knowledge, no similar approach has been reported in the literature to date.

## 2. Materials and Methods

### 2.1. Materials Used in the Study

Cement mortars investigated in this study were produced using Lafarge CEM I 42.5 R cement (Holcim Group, Bielawy, Poland). According to the manufacturer’s declaration, this cement complies with requirements of PN-EN 197-1:2002 [58], entitled “Cement—Part 1: Composition, specifications and conformity criteria for common cements.” The material is characterised by stable physicochemical properties, controlled setting behaviour, high early-age and final strength development, low alkali content, and enhanced resistance to chemical agents that may adversely affect the cement matrix. Thanks to those properties, the cement serves as a primary component ensuring phase stability, controlled hydration, and uniform microstructural development during mortar curing. Given the chemical and physical stability of this binder, it is possible to precisely observe thermal effects in the 100–600 °C range, including phase transformations within the cement matrix, depolymerisation of C–S–H gels, and thermal energy dissipation processes associated with the evaporation of free and chemically bound water. Detailed physicochemical parameters of the cement used are presented in Table 1.

The investigated mortar compositions also comprised Wisła sand with a 0/2 mm particle size distribution and Silimic microsilica (Silimic, Łaziska Górne, Poland). Silica fume, obtained as a secondary product during the manufacture of metallic silicon and ferrosilicon alloys in electric arc furnaces, is composed of extremely fine particles with an average size approximately two orders of magnitude smaller than that of Portland cement grains. According to the manufacturer’s specifications, partial replacement of cement with 15% microsilica results in a substantial improvement in mortar impermeability, providing several-fold lower permeability compared with conventional mixtures. This modification also contributes to a ca. 20% increase in compressive strength and a threefold reduction in water absorption. Nevertheless, when considering materials intended for high-temperature applications, the increased compactness of the cement matrix associated with silica fume incorporation may limit the elimination of moisture from capillary pores during heating. Water vapour expanding as a result of heating generates tensile stresses in the capillary walls, which, once the tensile strength is exceeded, may cause local damage and degradation of the composite. Nevertheless, in order to exploit the beneficial physicochemical effects of silica fume, it was decided to incorporate it into the investigated mortar composition. The basic properties of the microsilica, taken from the technical data sheet, are presented in Table 2.

Tap water meeting the requirements of PN-EN 1008:2004 [64] “Water for concrete—Specification for sampling, testing and assessing the suitability of water, including water recovered from processes in the concrete industry, as mixing water for concrete” was used for mortar preparation. The mixture was modified with Chrysofluid Optima 185 (Chryso Poland Ltd., Błonie, Poland), a chemical admixture compliant with the requirements of PN-EN 934-2 [65] “Admixtures for concrete, mortar and grout—Part 2: Concrete admixtures—Definitions, requirements, conformity, marking and labelling.” Chrysofluid is classified as a high-performance plasticising admixture, commonly applied in the production of high-strength and self-compacting concrete due to its ability to improve mixture workability. Its action is based on plasticisation and homogenisation of the mixture, ensuring time-dependent stability of consistency, as well as accelerating early cement hydration, which promotes faster development of early strength and a more uniform matrix microstructure. In the context of high-temperature studies, proper dispersion and uniform phase distribution in the presence of Chrysofluid enable improved dissipation of thermal energy, thereby reducing local thermal stresses. The basic properties of the applied admixture, according to the manufacturer’s technical data sheet, are presented in Table 3.

**Figure 1:**
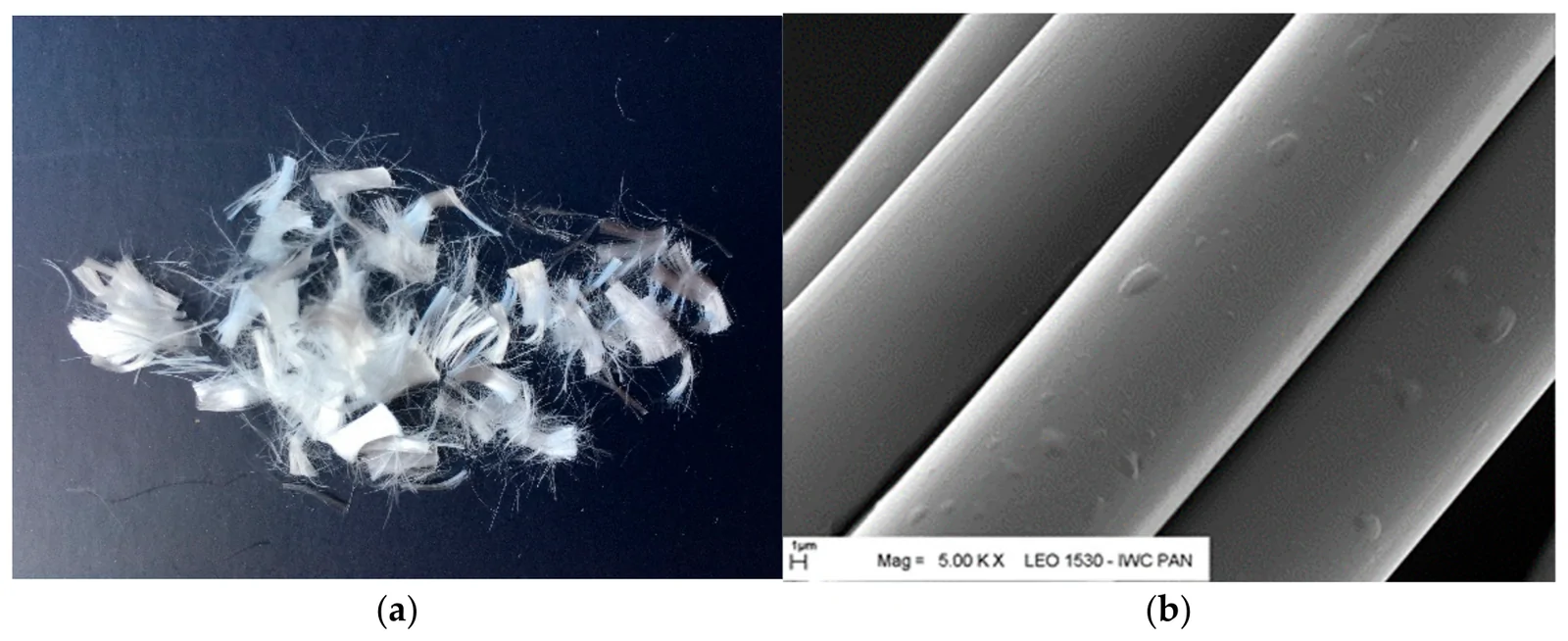
Polypropylene fibres I—(Ignis): (**a**) photograph without magnification; (**b**) SEM photo [47].

**Figure 2:**
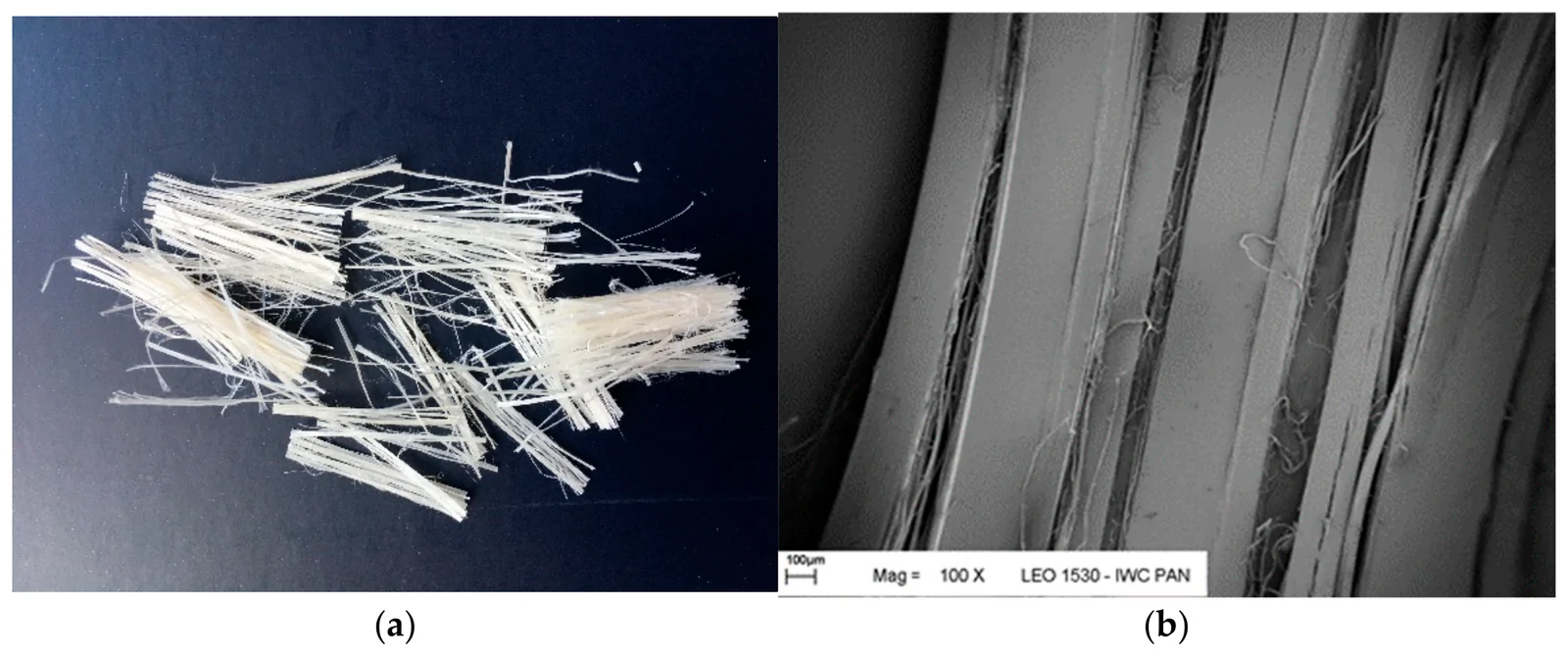
Polypropylene fibres F (Fibrofor Fibre High Grade 190): (**a**) macroscopic view; (**b**) SEM image.

Two types of polypropylene fibres, also used as concrete modifiers, were employed in the study and are designated herein as Type I and Type F (Figure 1 and Figure 2). Type I fibres are available for sale as Ignis^®^ (PP Nordica, Zgorzelec, Poland), whereas Type F fibres are supplied as Fortatech^®^ Fibre High Grade 190 (currently marketed as Fibrofor Fibre High Grade 190, Contec Fiber AG, Domat, Switzerland). The two fibre types differ primarily in terms of the length and diameter of individual filaments. Type I fibres are classified as monofilament fibres, with each fibre consisting of a single polypropylene filament. Type F fibres are multifilament, formed by twisting several thin filaments into a single structure. Both types of fibres act as dispersed micro-reinforcement, introducing elements with a length of ca. 12–19 mm and a diameter of 18–40 μm into the cement matrix. At high temperatures, these fibres contribute to the formation of pore channels after melting, which facilitates the release of water vapour from capillaries, reduces internal stresses, and limits spalling. Additionally, the presence of fibres contributes to the stabilisation of the matrix microstructure by modifying deformation mechanisms and improving thermal energy dissipation during heating.

The aim of introducing fibres was to reinforce the structure of unheated mortars, where they were intended to act as dispersed micro-reinforcement, forming a three-dimensional network within the concrete that increases its durability and tensile strength. During the setting phase, the fibres additionally reduce shrinkage-induced microcracks. Fibres were added to mortars subjected to elevated temperatures, with the aim of mitigating the effects of the limited capillary permeability of the cement matrix. Upon melting, the fibres generated additional pore channels that facilitated the controlled release of residual capillary water, thereby reducing the build-up of internal stresses and decreasing the risk of spalling. The fundamental characteristics of the fibres used in this study, based on the manufacturer’s data, are summarised in Table 4. The melting temperatures of the polypropylene fibres, determined by differential scanning calorimetry (DSC), were 161.8 °C for Ignis fibres and 164 °C for Fibrofor fibres [57].

The investigated material was a cement mortar with a low water-to-binder ratio (w/b = 0.23), and its structure further densified through the incorporation of silica fume. The addition of silica fume introduced highly reactive fine particles into the cementitious matrix, contributing to microstructural refinement by filling capillary pores and promoting additional hydration reactions. The composition of the reference mortar was provided by the manufacturer who uses it in the production of construction materials. The aim of this study was to evaluate the possibility of improving the mechanical performance of cement mortars exposed to elevated temperatures through the incorporation of polypropylene (PP) fibres into the mix. As part of the preliminary analyses, optimisation tests of tensile strength were conducted using fibres I and F at contents of 1.8 kg/m^3^, 3.0 kg/m^3^, and 3.6 kg/m^3^. The test results demonstrated that the addition of fibres I and F at the dosage recommended by the authors, i.e., 1.8 kg/m^3^, improved the mechanical performance at elevated temperatures. Based on the preliminary assessment of fibre performance, the mixture containing Type F polypropylene fibres at a dosage of 1.8 kg/m^3^ was selected as the reference composition for further mechanical testing. The selected fibre content was subsequently validated through experimental investigations presented in the following sections, as well as by a comparison with the results reported in earlier studies [56,57].

### 2.2. Sample Preparation

The test specimens were prepared at the Concrete Technology Laboratory of the Department of Building Materials Engineering, Faculty of Civil Engineering, Warsaw University of Technology. Prior to testing, cement mortar mixes were designed both without polypropylene fibre addition and with the incorporation of polypropylene fibres, maintaining identical constituent proportions within each experimental series. Specimens in each test series were produced using an identical mix composition as the one given in Table 5 to ensure consistency of the microstructure and allow a reliable assessment of the effect of fibre incorporation on thermal energy dissipation and the preservation of mechanical properties under elevated-temperature conditions.

All mortar mixtures have been prepared using a laboratory mixer. The procedure began with accurate weighing of the dry materials, namely cement and sand, followed by their dry mixing for approximately 60 s to ensure an even distribution of the solid phases throughout the mixture. Subsequently, polypropylene fibres were added and mixed for 30 s; the short mixing time was due to the tendency of the fibres to agglomerate and adhere to the mixer blades during prolonged mixing. The subsequent stage involved the addition of water and a plasticiser at a dosage of 2% of the cement mass, after which all components were mixed for approximately 3 min, ensuring uniform fibre dispersion and complete distribution of capillary water within the cementitious matrix.

The specimens were formed by casting the mortar into moulds in two successive layers, followed by vibration for approximately 15 s to minimise the development of macropores and promote a uniform internal structure. After casting, the specimens were kept on a level laboratory surface and sealed with plastic foil to prevent excessive evaporation of moisture from the exposed surfaces. Following a 24 h period, the samples were removed from the moulds and subjected to water curing for 27 days to allow the cement hydration process to proceed and facilitate the formation of the C–S–H gel structure. The cured specimens were then stored in a climatic chamber at 20 °C and 99% relative humidity for 60 days to achieve moisture equilibrium within the cement matrix before drying.

To prepare the specimens for thermal testing, the elements were dried in a laboratory oven at 70 °C to a constant mass for 21 days, while water loss was continuously monitored. The drying process minimised the risk of violent explosions caused by boiling capillary water in specimens not modified with PP fibres during heating to 600 °C, which could otherwise damage the testing equipment. An individual identification code was assigned to each specimen, and the casting date was duly specified to enable unambiguous identification and correlation with the recorded test data. All the samples were manufactured and conditioned under the same environmental conditions prior to testing. For each test series, five mortar specimens without PP fibre reinforcement and five specimens incorporating F-type polypropylene fibres were prepared to provide statistical reliability of the obtained results.

### 2.3. Methods

The compressive strength used for mortar classification was determined using 100 × 100 × 100 mm cubic specimens. Flexural strength was evaluated on prismatic specimens measuring 40 × 40 × 160 mm, while compressive strength following flexural failure was established using the two prism halves remaining after the bending test. The tensile modulus of elasticity was determined using “dog-bone”-shaped specimens. Such specimen geometries made it possible to assess the mechanical integrity of the cementitious matrix both under conditions of uniform laboratory temperature and those involving non-uniform thermal stress distributions. Moreover, after the destruction of the samples, it was possible to observe the effect of microstructural changes that had occurred during heating.

**Figure 3:**
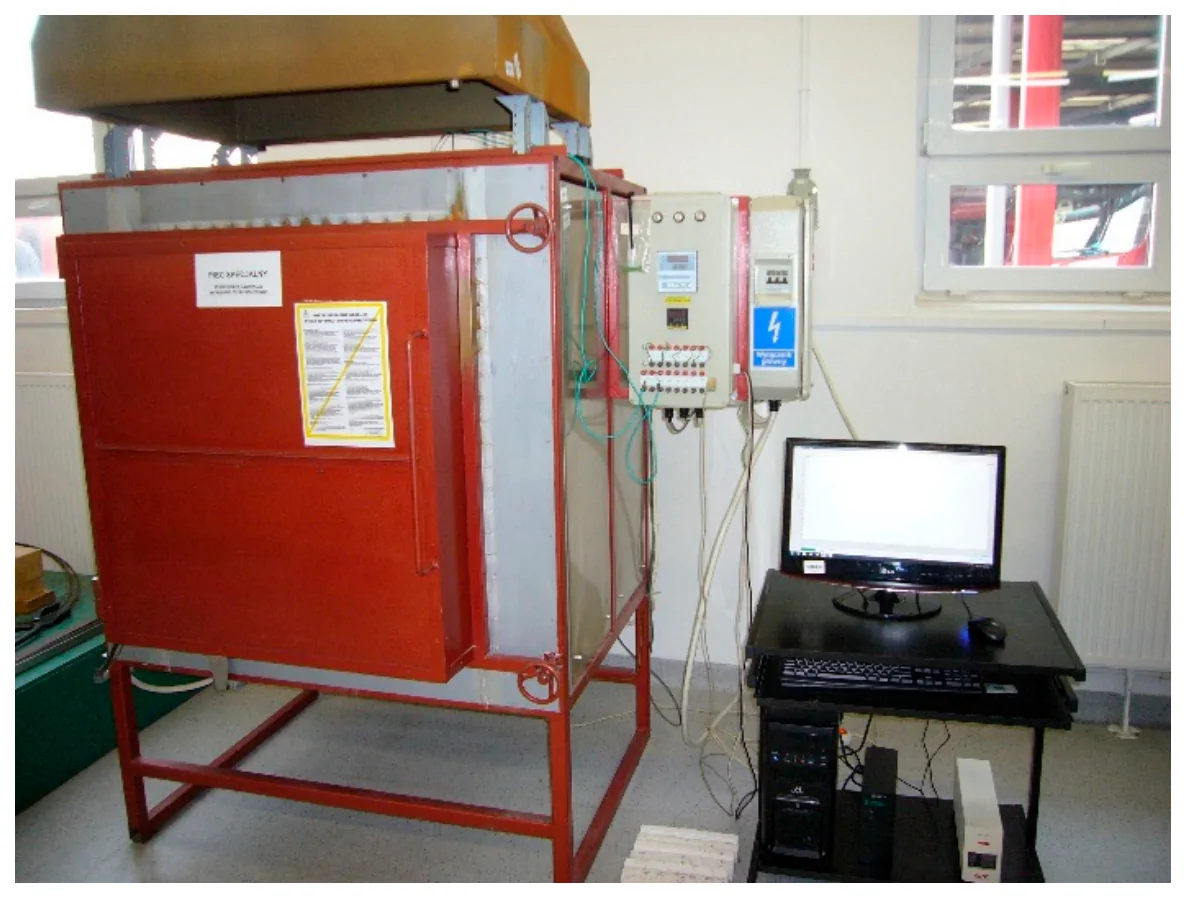
Sample annealing station [56].

The simulation of high-temperature exposure was carried out at the Laboratory of Applied Mechanics of the Academy of Fire Service. Specimens were heated in an electric chamber furnace, model PK 1100/5, coupled with a computer-based control system (Figure 3). The heating process was monitored, and temperature changes were recorded in real time using specialised software developed by Thermolab S.C. (Warsaw, Poland). The ThermoPro v. 1.2 SGSP software allows the user to define customised temperature profiles throughout the test duration, including the setting of constant temperature stages for specified time periods and the regulation of the heating rate until the required temperature level is achieved. This made it possible to accurately reproduce the temperature profile and monitor heat energy dissipation within the specimen.

After completion of the designated curing period, the specimens of each composite type were divided into groups subjected to thermal loading. Test temperatures covered the range of 20 °C to 600 °C. The upper temperature limit adopted in the experimental programme was defined based on the range in which the contribution of polypropylene fibres to the behaviour of the cementitious matrix could be evaluated. In this temperature range, it was possible to monitor PP fibre degradation, the formation of capillary channels in their place, and to observe how these structures contribute to the dissipation of water vapour energy within the pores and to the reduction in internal stresses.

According to earlier studies [57], above 600 °C, the polypropylene fibres undergo complete decomposition, and their direct reinforcing and compensating effect is no longer observable. Consequently, the experimental temperature range was defined up to this maximum value. The specimens were finally exposed to furnace heating at six temperature stages, namely 100 °C, 200 °C, 300 °C, 400 °C, 500 °C and 600 °C. During the experiments, efforts were made to maintain a temperature–time profile as close as possible to standard fire conditions, so as to reproduce the actual thermo-physical and phase transformation processes occurring within the cementitious matrix, including evaporation of physically and chemically bound water, depolymerisation of C–S–H gels, and gradual rehydration of residual CaO.

Temperature monitoring during the heating procedure was performed with the use of three thermocouples: one reference thermocouple (TR) for recording the furnace temperature and two additional sensors (T1 and T2) for measuring temperature inside the specimen. Thermocouple T1 was positioned on the specimen surface, whereas thermocouple T2 was installed inside a previously drilled channel. Its end was located at mid-thickness of the specimen. This setup enabled simultaneous recording of the temperature gradient across the entire specimen volume and allowed for the assessment of heat energy dissipation within the cementitious matrix.

For every selected temperature level, the prepared specimen sets were placed in the furnace together with an additional reference specimen equipped with thermocouples T1 and T2 for monitoring of the internal temperature. The heating process was compliant with the recommendations of PN-EN 1991-1-2 Eurocode 1: Actions on structures–Part 1-2: General actions–Actions on structures exposed to fire [66] (Polish Committee for Standardisation, Warsaw, 1991). Throughout the experiment, the temperature–time relationship was controlled to reproduce, as closely as possible, the thermal exposure conditions corresponding to a standard fire. The heating cycle and temperature acquisition, with a recording frequency of up to 1 Hz, were controlled using dedicated Thermolab software (version of the software: ThermoPro v. 1.2 SGSP). The ThermoPro software provides the capability to define customised temperature–time profiles throughout the test, maintain selected temperature levels for predetermined durations, and regulate the heating rate until the required temperature is achieved. Temperature measurements were performed using Type K thermocouples (NiCr–NiAl) compliant with PN-EN 60584-1:2014-04 [67]. This standard specifies the relationship between the measured temperature and the thermoelectric voltage generated by individual thermocouple types. The specimens were heated until the predefined temperature level was reached, based on the readings provided by the thermocouples, which required approximately 120 min. Once the temperature had stabilised, it was maintained for further 30 min to ensure full thermal homogenisation of the specimen and to initiate phase transformation processes within its structure. Following completion of each heating stage, the furnace was deactivated and allowed to cool to approximately 100 °C. Subsequently, the specimens were subjected to uncontrolled cooling under laboratory conditions for 24 h until thermal equilibrium with the surroundings was achieved (20 °C).

**Figure 4:**
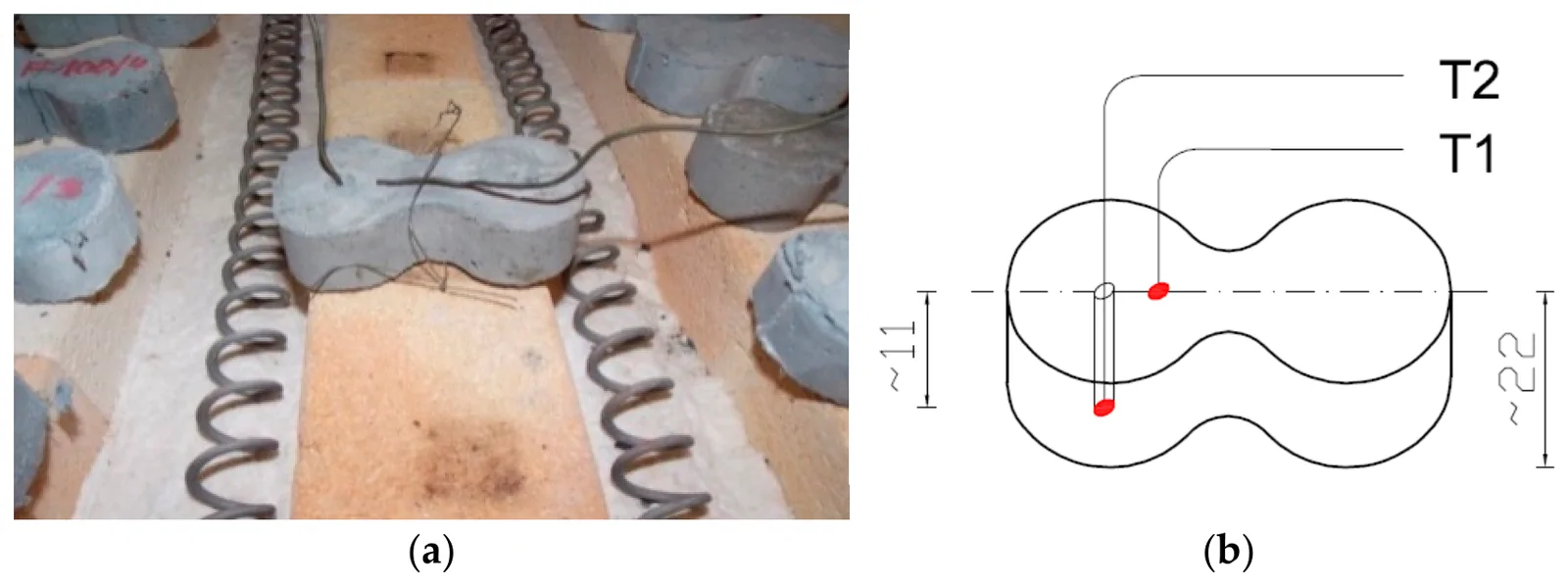
Configuration of thermocouples in the “figure-eight” specimen: (**a**) view of the specimen with attached thermocouples; (**b**) schematic representation of thermocouple locations within the specimen [56].

The initial phase of the research, consisting of optimisation trials, included tensile strength measurements performed on “figure-eight”-shaped specimens. As in the previously described temperature-monitoring procedure, each heating series included, apart from the reference specimen equipped with thermocouples, additional groups of specimens containing two types of polypropylene fibres incorporated at dosages of 1.8 kg/m^3^, 3.0 kg/m^3^, and 3.6 kg/m^3^ [57]. The prepared batches of specimens were subjected to heating at six different temperatures: 100 °C, 200 °C, 300 °C, 400 °C, 500 °C and 600 °C. The reference temperature was set at 20 °C (Figure 4a,b). This approach made it possible to monitor the effect of increasing temperature on fibre structure, the formation of capillary channels after fibre melting, and the resulting consequences for the mechanical response and energy dissipation within the composite.

**Figure 5:**
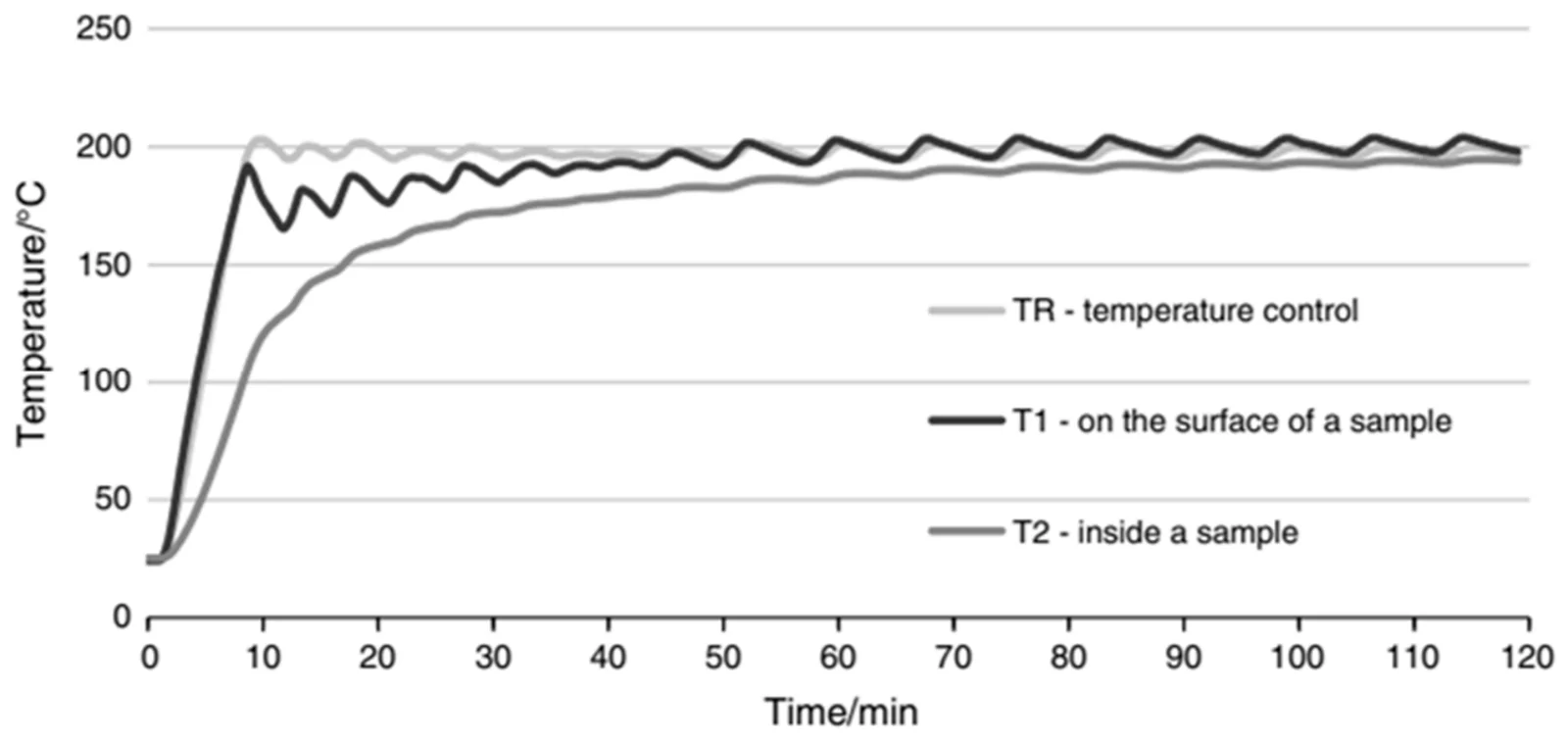
Example of heat treatment of the mortar sample at 200 °C [68].

Figure 5 shows an example of the curve illustrating the actual temperature distribution in an “eight-shaped” specimen at the locations of the measurement thermoelements [68].

Within the optimisation programme, an appraisal was performed of the tensile strength of “dog-bone”-shaped specimens. The tests were conducted in compliance with PN-85 B-04500 [69] “Building mortars—Testing of physical and mechanical properties.” An INSTRON 5567 universal testing machine (INSTRON, Norwood, MA, USA) was used for the measurements, with a load capacity of 0–30 kN and equipped with specially designed curved grips to ensure reliable fixation of the specimens during testing. The “dog-bone” specimens were manufactured using dedicated detachable steel moulds prepared in accordance with the requirements specified in the standard.

**Figure 6:**
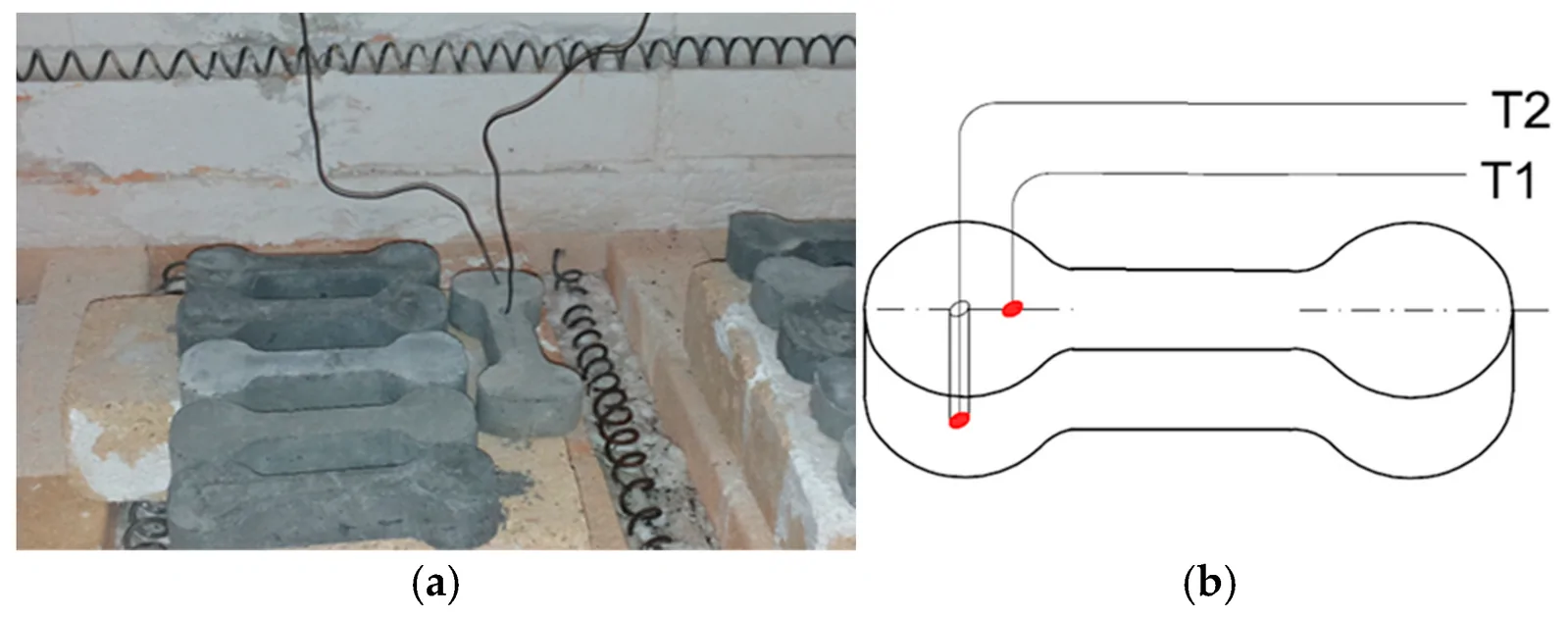
Arrangement of measuring thermocouples in an elongated dog-bone-shaped specimen: (**a**) view of the sample with thermocouples attached; (**b**) diagram of the arrangement of thermocouples in the sample.

**Figure 7:**
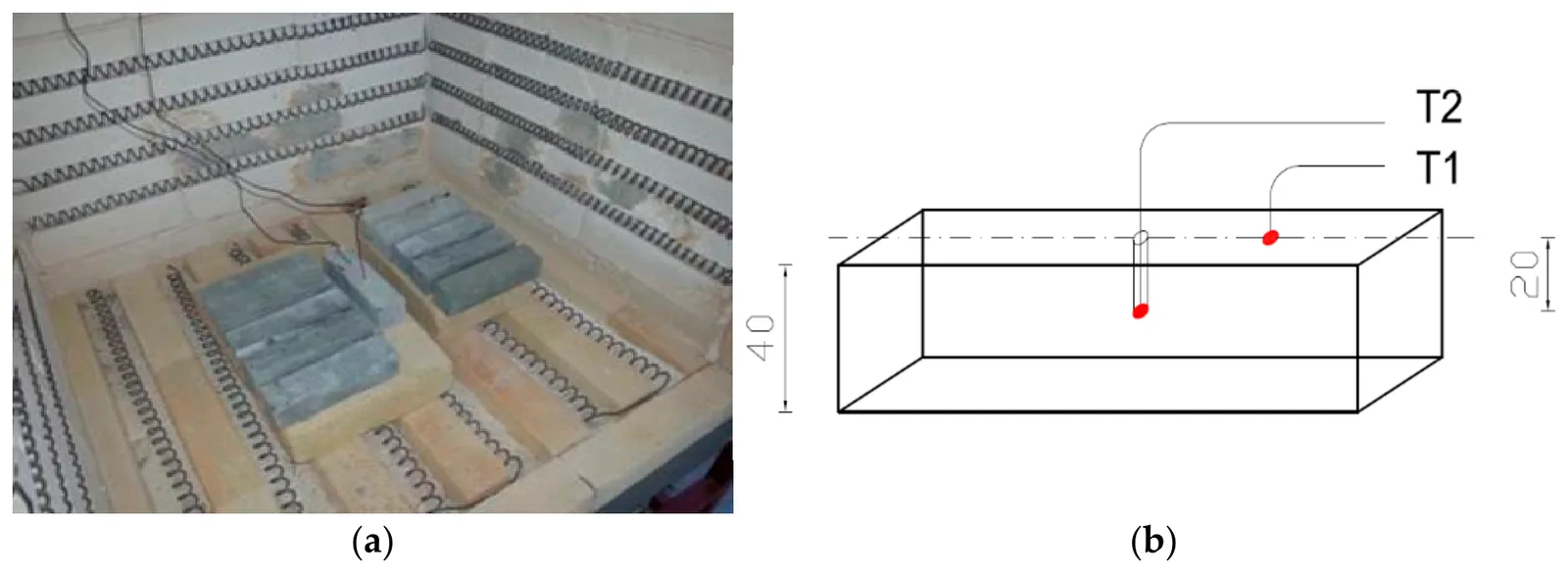
Arrangement of measuring thermocouples in a specimen with nominal dimensions of 40 × 40 × 160 mm: (**a**) view of the specimen with mounted thermocouples; (**b**) schematic layout of thermocouples within the specimen [56].

Figure 6 and Figure 7 show a view of the furnace chamber together with the arrangement of specimen batches intended for testing the modulus of elasticity on “dog-bone”-shaped specimens and flexural strength on prismatic specimens with nominal dimensions of 40 × 40 × 160 mm. This range of mechanical tests was performed on an increased number of specimens (five specimens per measurement series). Each time, in addition to the specimen equipped with thermocouples T1 and T2, a total of ten specimens were subjected to each heating cycle, comprising five unmodified mortar specimens and five specimens reinforced with polypropylene fibres at 1.8 kg/m^3^.

Mechanical testing was conducted at the Department of Building Materials Engineering, Warsaw University of Technology. The INSTRON 5567 universal testing machine was used to determine the modulus of elasticity of “dog-bone”-shaped specimens and to assess the flexural strength and subsequent compressive strength of the fractured mortar specimens (INSTRON, Norwood, MA, USA). This specimen preparation procedure ensured a homogeneous cementitious matrix microstructure and minimised local stresses resulting from imperfections in moulding, which is crucial for assessing energy dissipation.

**Figure 8:**
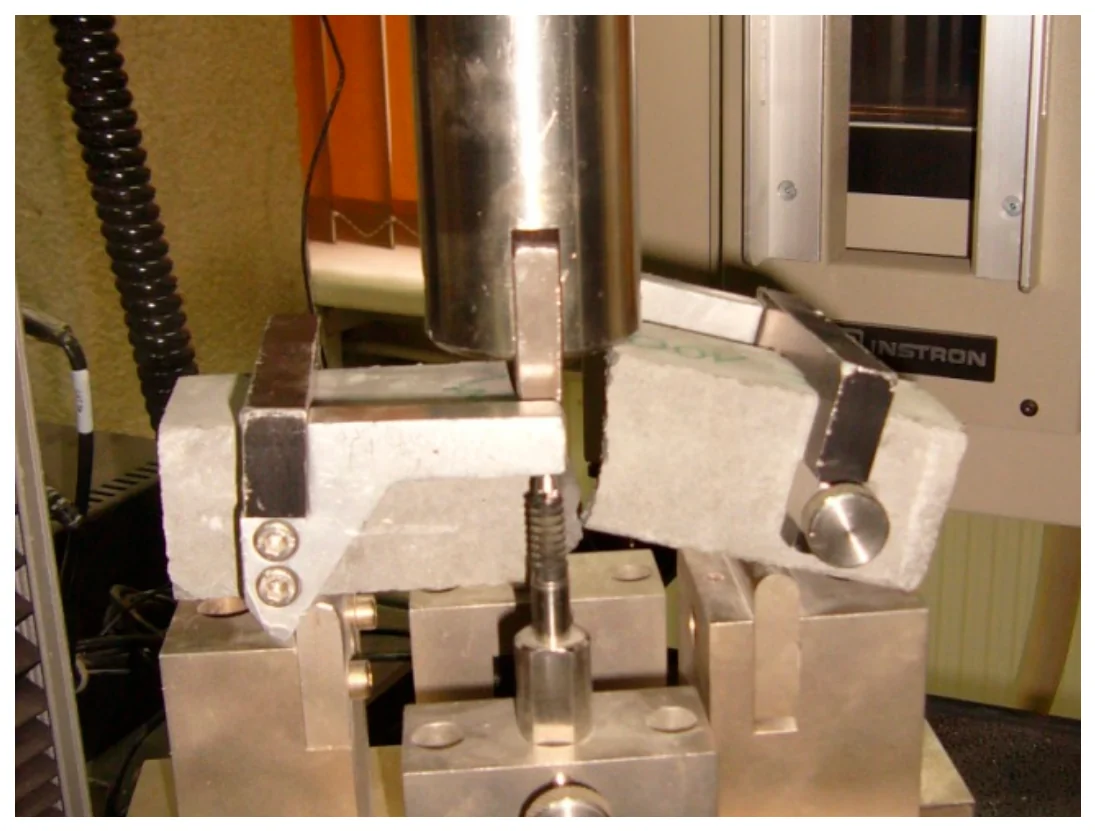
Station for determining flexural strength on 40 × 40 × 160 mm beams.

The flexural strength of the mortar (Figure 8) and the compressive strength (after fracture) were determined on prismatic specimens with nominal dimensions of 40 × 40 × 160 mm, according to the procedure specified in standard [70] PN-EN 1015-11:2001 “Methods of test for mortar for masonry—Part 11: Determination of flexural and compressive strength of hardened mortar.”

The reference compressive strength (fc) of the mortar, required for strength-grade determination, was measured on cubic specimens (100 × 100 × 100 mm) that had not been subjected to thermal exposure. The measurements were performed according to PN-EN 998-2:2016-12 [71] “Specification for mortar for masonry—Part 2: Masonry mortar.” This procedure enabled the determination of the mortar grade. In the case of cement mortars with and without polypropylene fibre addition, a suitably adapted CONTROLS MCC8 testing machine was deployed for the testing (CONTROLS GROUP, Liscate (Milan), Italy).

**Figure 9:**
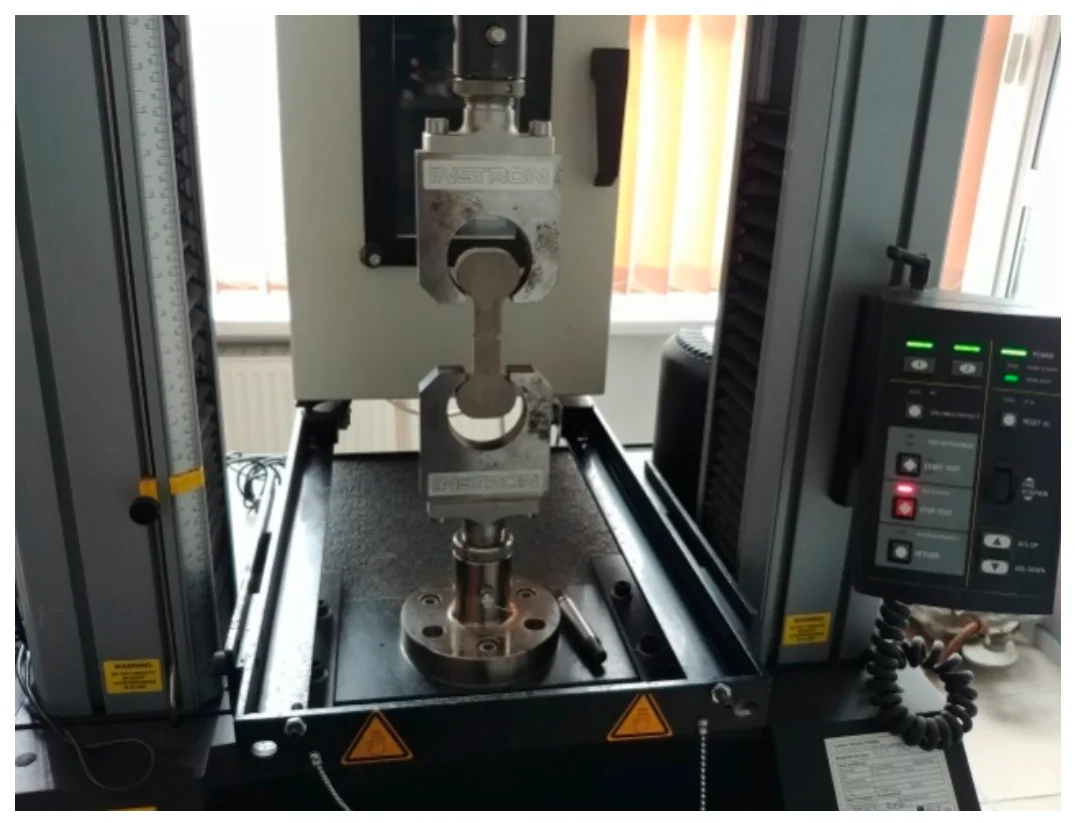
Station for determining the tensile modulus of elasticity used on samples shaped like “elongated dog bones”.

The modulus of elasticity (Figure 9) was determined on “dog-bone”-shaped specimens using a specially adapted INSTRON 5567 testing machine (INSTRON, Norwood, MA, USA) equipped with curved grips for specimen mounting, and a measurement range of 0–30 kN.

In the context of cement mortars, this test enabled not only the determination of material stiffness (i.e., its resistance to deformation under load within the elastic range), but also an indirect assessment of microstructural changes in the cementitious matrix induced by exposure to high temperatures.

## 3. Results

**Figure 10:**
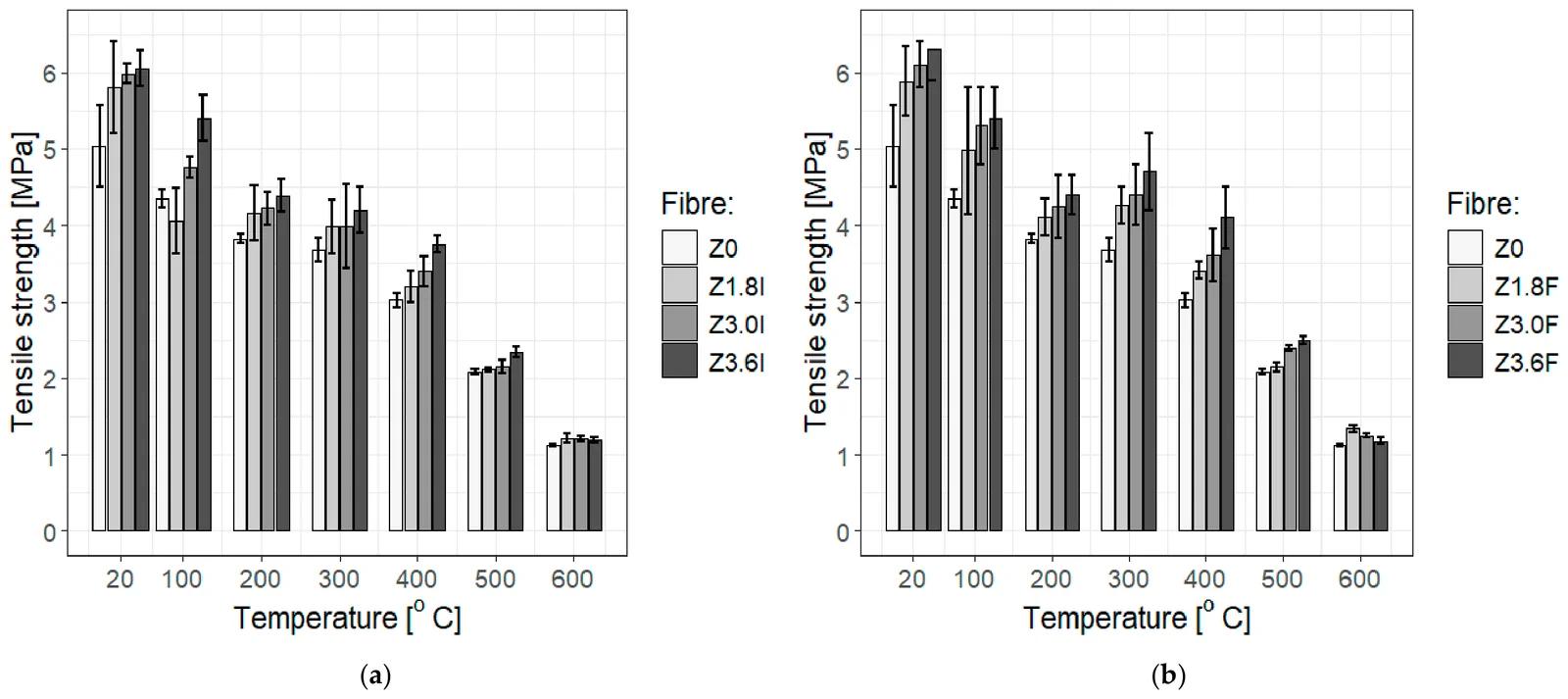
Mean tensile strength ± standard deviation of mortar specimens without PP fibres (Z0) and with PP fibres at different fibre contents: Z1.8x (1.8 kg/m^3^), Z3.0x (3.0 kg/m^3^), and Z3.6x (3.6 kg/m^3^), where x = F or I; (**a**) polypropylene fibres I (Ignis); (**b**) polypropylene fibres F (Fibrofor Fibre High Grade 190) [57].

As part of the preliminary analyses, optimisation tests of tensile strength were conducted using fibres I and F at dosages of 1.8 kg/m^3^, 3.0 kg/m^3^, and 3.6 kg/m^3^ [56,57]. The conducted tests also confirmed more favourable mechanical performance for fibre F (Figure 10).

The experimental findings indicated that both the exposure temperature and the type of polypropylene fibre exerted a significant influence on the mechanical performance of the mortars. Irrespective of mixture composition, strength was found to progressively decline as the temperature increased. Nevertheless, the incorporation of fibres generally reduced the extent of thermal deterioration and contributed to higher tensile strength. As illustrated in Figure 10a,b, polypropylene fibre reinforcement enhanced the strength of the cement mortars across a broad range of temperatures. Under ambient conditions (20 °C), specimens containing either fibre type exhibited an approximate 16% increase in strength relative to the unreinforced control mortar, confirming the beneficial contribution of dispersed fibre reinforcement prior to thermal exposure. The performance was found to have been considerably improved within the temperature interval of 200–400 °C, in which the presence of polypropylene fibres was found to be particularly advantageous. While both Ignis and Fibrofor fibres enhanced tensile strength with respect to unreinforced mortar, this effect was found to be more evident in mixtures incorporating Fibrofor fibres. According to these results, fibre reinforcement allows the mechanical properties of mortar to be partially retained under conditions of moderate thermal loading. However, once the temperature was higher than 500 °C, all the samples had a similar response–their tensile strength fell by more than five times. Regardless of this significant decline in the properties of the fibre-reinforced mortar, the samples demonstrated continuously slightly higher tensile strength than the reference samples. The improved strength was ca.5–7% for Ignis fibres and 5–10% for Fibrofor fibres at respective dosages of 3.6 and 3.0 kg/m^3^, rising to almost 19% for mortar incorporating 1.8 kg/m^3^ of Fibrofor fibres.

The results of these tests have shown that the addition of I and F fibres at the dosage recommended by the authors (1.8 kg/m^3^) may allow reducing the spalling phenomenon taken into account in the tested conditions. Consequently, the composition of the reference mortar intended for further mechanical testing was modified by incorporating F-type fibres at a dosage of 1.8 kg/m^3^.

**Figure 11:**
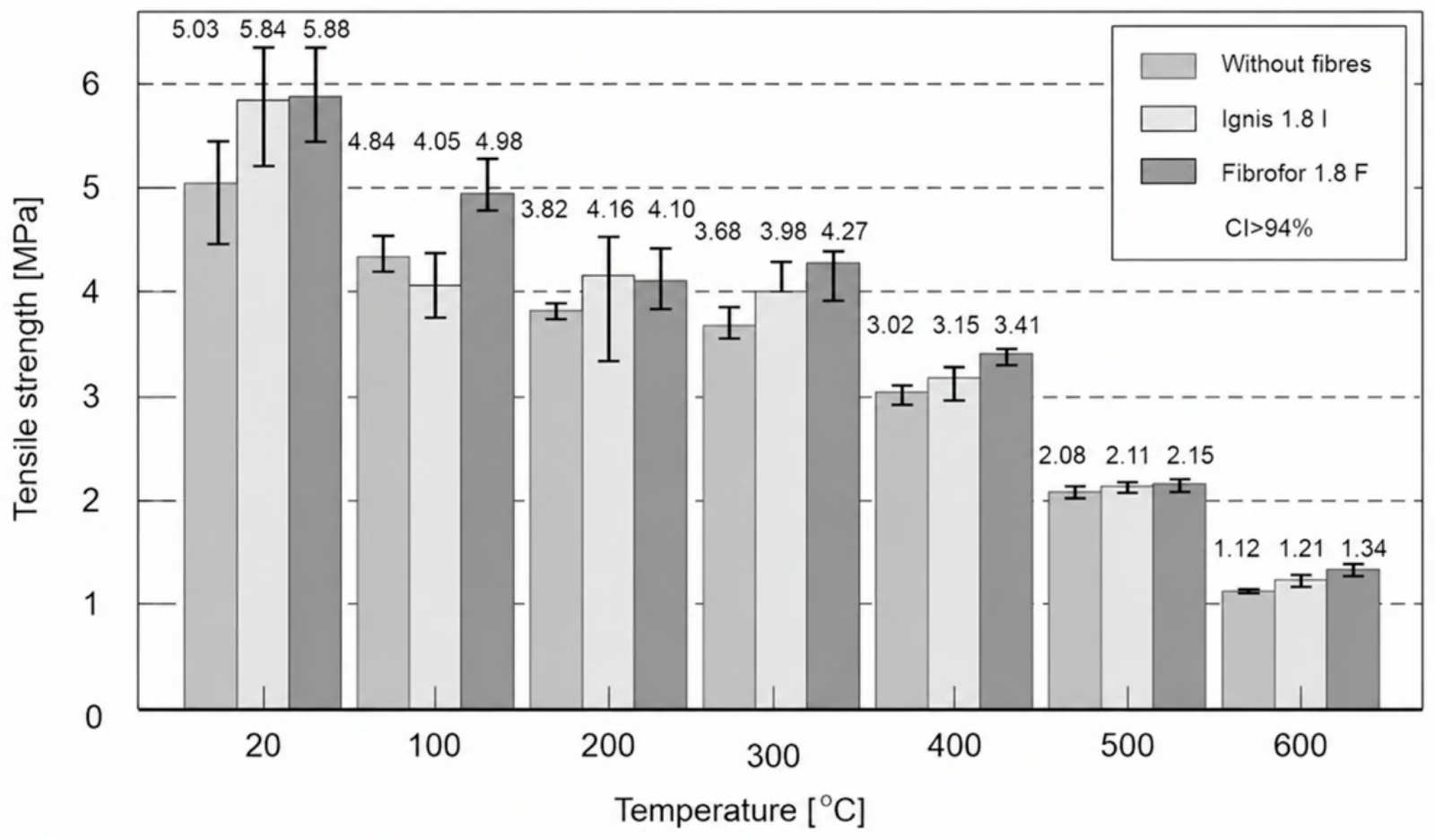
Tensile strength of figure-eight specimens containing different fibre types—results of the optimisation study [56].

The results of the optimisation study—tensile strength tests performed on different types of fibres, which were conducted on eight samples—are presented in Figure 11 [56].

A review of the obtained results demonstrates a clear influence of both exposure temperature and the type of polypropylene fibres used on the development of mechanical properties of the mortar [56]. The role of polypropylene fibres on mechanical properties of fibre-reinforced mortars at various temperatures can be explained as follows. Regardless of the mix composition, an increase in temperature leads to a gradual degradation of strength parameters, which can be attributed to progressive dehydration of cement hydration products, reorganisation of the C–S–H gel, and increasing strain incompatibility between phases of the composite. In parallel, in most cases the presence of fibres partially compensated for these adverse effects by modifying the mechanisms of stress transfer and strain energy dissipation. Under ambient conditions (20 °C), mortars reinforced with either type of polypropylene fibre exhibited an approximately 16% higher tensile strength than the unreinforced reference specimens. This effect confirms the beneficial role of dispersed reinforcement in the initial state, which consists of limiting the initiation and propagation of microcracks in the cementitious matrix and increasing the material’s ability to absorb elastic energy.

A different behaviour was observed after exposure of the specimens to a temperature of 100 °C. In the series with Ignis fibres, a decrease in strength of ca. 7% was recorded as compared to the reference specimens, whereas in the case of Fibrofor fibres a clear increase in strength was observed—up to ca. 15% relative to the fibre-free mortar. At this stage, a distinct differentiation in the material response to thermal exposure depending on the type of fibre used became evident. Since no substantial thermal degradation of polypropylene occurs within this temperature range, the observed differences should primarily be attributed to the distinct fibre morphology and its interaction with the cementitious matrix. F-type fibres, characterised by a multifilament structure, provide a more developed contact surface with the cement paste, which promotes improved interfacial adhesion and more effective stress transfer. From a chemical perspective, this implies stronger physicochemical interactions at the fibre–matrix interface, while from a mechanical standpoint it results in a more uniform energy dissipation within the microstructure of the composite.

In the temperature range of 200–400 °C, the influence of fibres on mechanical properties of the mortar was found to be clearly beneficial. Both fibre types led to an increase in strength as compared to the reference specimens; however, this effect was markedly more pronounced for Fibrofor fibres. The greatest differences were observed at 300 °C, where the strength increase reached approximately 16% for F-type fibres and only about 8.5% for I-type fibres. A similar trend was noted at 400 °C, where the advantage of multifilament fibres persisted, although its magnitude was smaller. In this temperature range, intense chemical processes occur within the cementitious matrix, including the breakdown of hydration bonds, partial depolymerisation of the C–S–H gel, and dehydroxylation of portlandite. The presence of fibres reduces the stress concentration generated by these transformations by acting as crack-bridging elements at the microcrack level and enhancing the material’s ability to dissipate thermo-mechanical energy.

Beyond 500 °C, the mechanical response of all mortar series became increasingly similar, suggesting that extensive thermal damage to both the cementitious matrix and the polypropylene fibres had largely eliminated the beneficial effect of fibre reinforcement. However, even within this range, fibre-reinforced specimens exhibited more favourable mechanical performance than the unreinforced reference mortar. This was particularly evident at 600 °C, where the use of Fibrofor fibres enabled the retention of a compressive strength approximately 20% higher compared to the reference mortar. Ignis fibres also demonstrated a positive effect, although of lower magnitude (approximately +8%).

Overall, the experimental results demonstrate that dispersed polypropylene fibre reinforcement effectively improves the resistance of cement mortars to elevated temperatures. Among the two fibre types investigated, multifilament Fibrofor fibres consistently delivered superior performance throughout the entire temperature range, contributing to both higher initial tensile strength and a more stable mechanical response during thermal exposure. Although the incorporation of Ignis fibres also enhanced the mechanical behaviour of the mortars, their beneficial effect was less consistent, particularly at lower exposure temperatures.

After establishing the composition of reference mortar intended for further testing, compressive strength (fc) tests were carried out to establish the mortar grade. The tests were performed on cubic specimens with nominal dimensions of 100 × 100 × 100 mm at a temperature of 20 °C, in accordance with standard [71] PN-EN 998-2:2016-12 “Specification for mortar for masonry—Part 2: Masonry mortar.” The tests were conducted with the use of a CONTROLS MCC8 testing machine in accordance with the procedure described in [72] PN-EN 12390-3:2011 “Testing hardened concrete—Part 3: Compressive strength of test specimens.” The results of compressive strength tests for cement mortars without fibre addition and those modified with Fibrofor polypropylene fibres at a dosage of 1.8 kg/m^3^ are presented in Table 6 and Table 7. The compressive strength results (fc) were obtained on specimens not subjected to thermal loading at 20 °C after 28 and 90 days of conditioning in a climatic chamber at a relative humidity of RH = 99% and a temperature of 20 °C. The reported results represent the arithmetic mean of measurements performed on three specimens.

**Figure 12:**
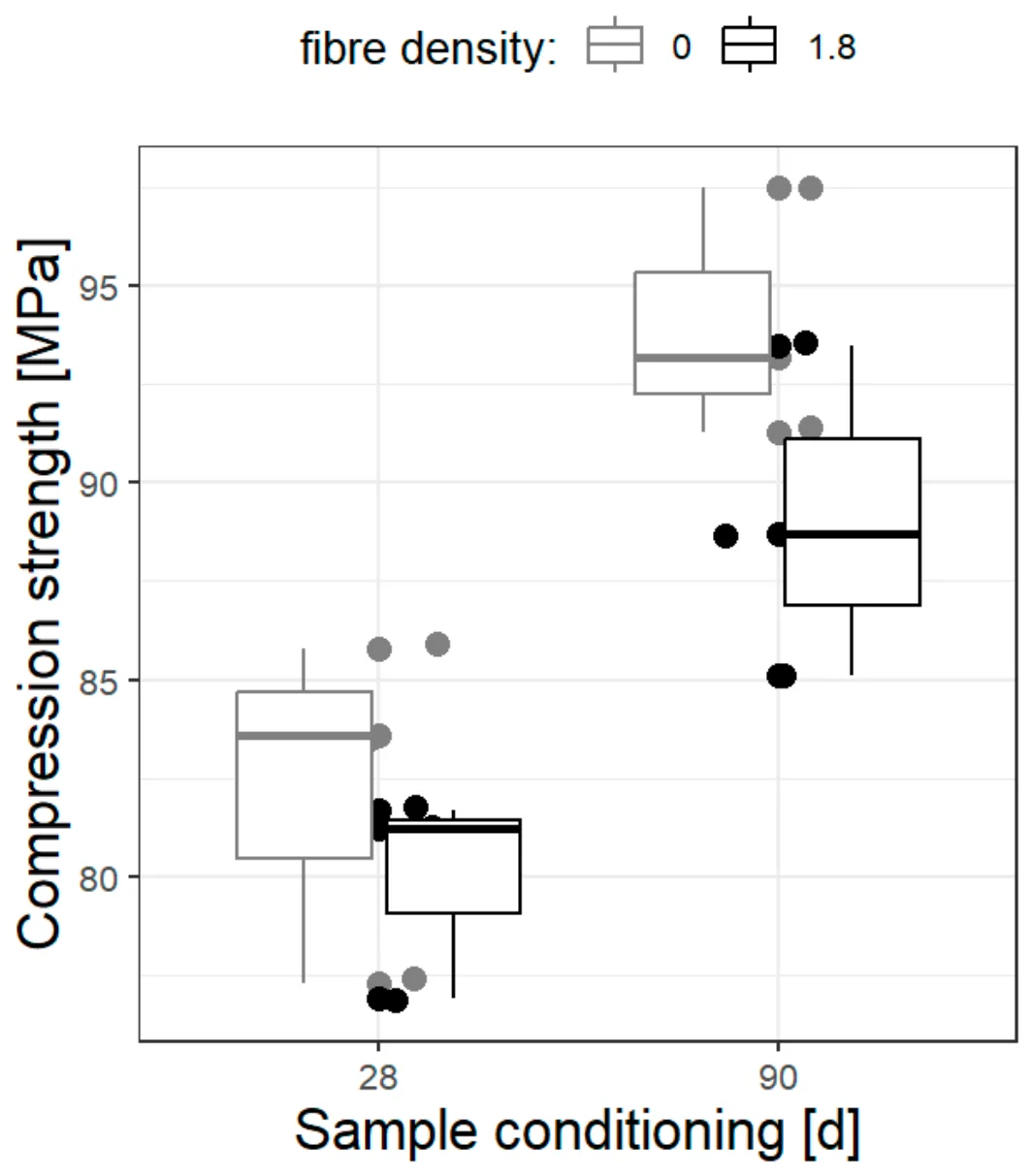
Statistical box-plots of compressive strength [MPa] as dependent on days of ageing of mortar reference samples (grey), and mortar samples reinforced with PP fibres (black). After 90 days of conditioning the compressive strength of samples with PP fibres is lesser than in the case of mortar with no added fibre.

Based on the obtained results (for specimens not subjected to thermal loading at 20 °C), it was confirmed that the addition of polypropylene fibres to cement mortars (Z1.8F) led to a reduction in the tested strength parameter compared to the conventional cement mortar specimens (Z0F). The results for specimens conditioned for 28 and 90 days also confirm a decrease in the compressive strength of cement mortars containing polypropylene fibres. The difference in compressive strength after 28 days of conditioning is approximately 2.3 MPa, while after 90 days it reaches nearly 5 MPa. The curing period of the specimens had a significant influence on the obtained results. For both unmodified mortar specimens (Z0F) and those containing fibres (Z1.8F), the difference in results is approximately 10 MPa (Figure 12).

These results have been partially confirmed by the statistical analysis presented in Table 8, which comprises descriptive statistics and statistical test outcomes. A comprehensive review of statistical analysis methods can be found in [73]. All plots and analyses were performed using the R software environment (version of the software: 4.6.1.), for which full documentation is available in [74].

A correct comparative analysis of means across two datasets is typically performed in three steps: verification of distribution normality (Shapiro–Wilk test—however, a distribution can be considered normal when the *p*-value is greater than the adopted significance level α, most commonly set at 0.05), assessment of variance homogeneity (e.g., Fisher’s test; if the distributions are normal, then, as before, *p* > α indicates that the null hypothesis of equal variances in both datasets cannot be rejected), and finally the application of an appropriate comparison test—parametric if the distributions are normal, or non-parametric if they deviate from normality. In this case, the compressive strength results satisfied both criteria (normality and homogeneity of variances), therefore a simple *t*-test could be performed (last column in Table 8). The interpretation is as follows: if *p* < α, the null hypothesis should be rejected in favour of the alternative one, which in this case states that the mean compressive strength of mortar without fibre addition is statistically higher than that of the PP fibre-reinforced mortar. Table 8 allows the conclusion that this relationship holds with a probability of 90%. In other words, for specimens conditioned for 90 days, it can be stated at the 90% confidence level that the compressive strength of F-fibre-reinforced mortars is statistically significantly lower than that of mortars without fibre addition. For the shorter conditioning period (28 days), the inference is less straightforward. A *p*-value of 0.24 indicates that a similar conclusion could only be drawn at a significance level of approximately 75%, which is considered too low to be statistically meaningful. In summary, it is evident that conditioning increases the compressive strength of mortars; however, this effect is less pronounced in fibre-reinforced mortars than in those without fibres.

The reduction in compressive strength of cement mortars modified with polypropylene fibres is caused by the fact that these fibres introduce discontinuities within the matrix structure, which contributes to a decrease in material cohesion. Previous studies [6] on fibre-modified cement pastes (CEM I 42.5 R and CEM I 52.5 R) also demonstrate a reduction in compressive strength compared to fibre-free cement paste specimens, regardless of the presence of a Pozzolanic addition.

Another test performed on specimens considered optimal was the flexural tensile strength test. For cement mortars, this test is a parameter that is particularly sensitive to microstructural changes induced by temperature, as its value depends to a great extent on the integrity of the cementitious matrix, the continuity of the binding phases, and the characteristics of the interfacial transition zones (ITZ). In this study, flexural strength analysis was conducted on prismatic specimens measuring 4 × 4 × 16 cm, made of both reference mortars and mortars modified with F-type polypropylene fibres at a dosage of 1.8 kg/m^3^.

**Figure 13:**
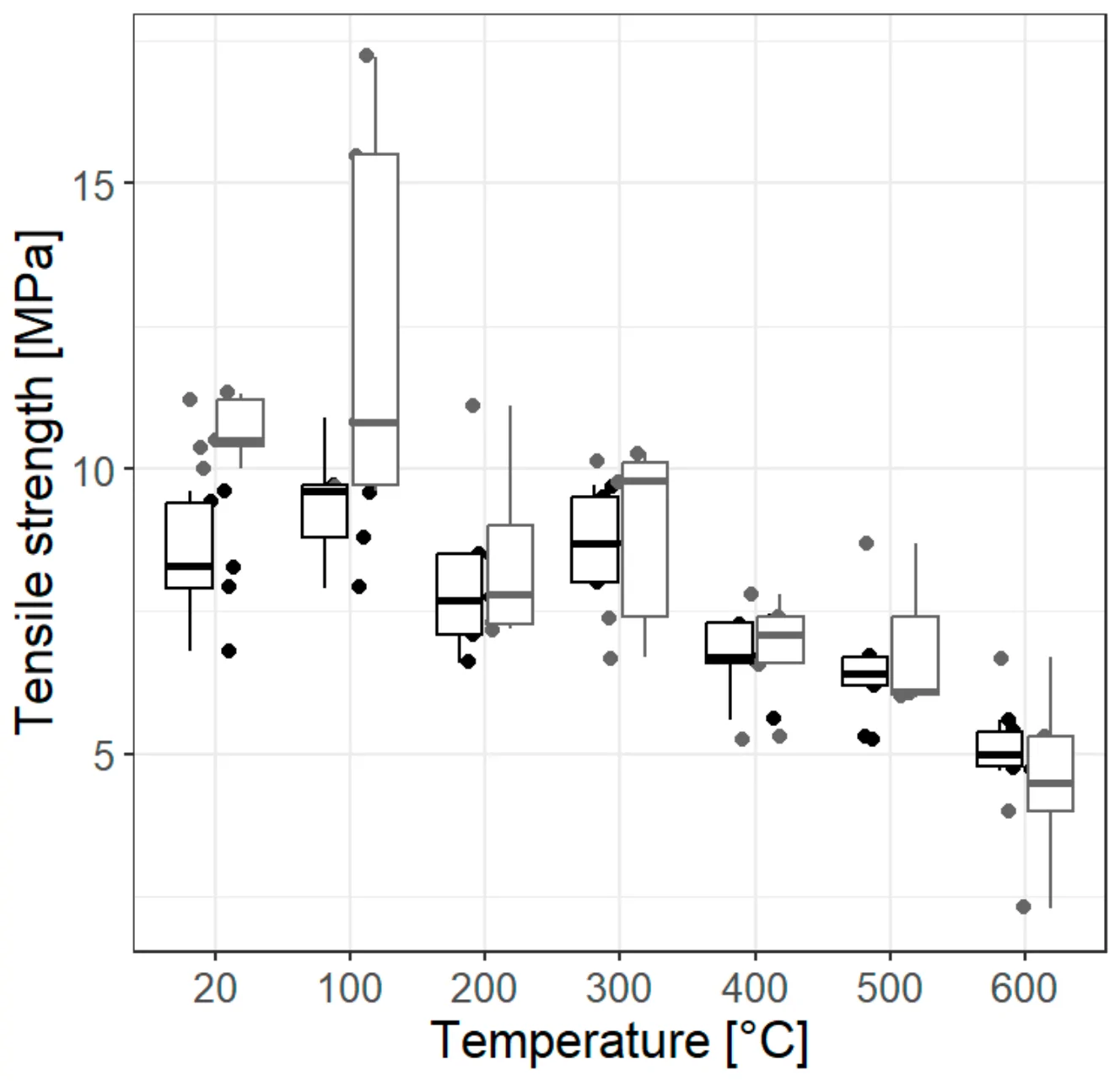
Flexural tensile strength of mortar specimens without fibre addition (black) and with PP fibre addition at a dosage of 1.8 kg/m^3^ (grey). A box-and-whisker plot has been superimposed on the scatter plot (raw data), where the box represents the interquartile range (from the first to the third quartile), the horizontal line indicates the median, and the whiskers denote the minimum and maximum values within the sample. The data points were slightly jittered for improved visual clarity. After an initial period of relatively constant strength, above 300 °C the value continuously decreases with increasing temperature.

The relationship between flexural tensile strength and temperature for prismatic specimens (4 × 4 × 16 cm) made of reference mortars and mortars modified with F-type polypropylene fibres is presented in Figure 13.

In this study, both for reference mortars and polypropylene fibre-reinforced mortars, a slight increase in flexural tensile strength was observed up to 100 °C, followed by a relatively broad plateau extending to approximately 300 °C, and a subsequent decrease in strength at higher temperatures. For unheated specimens, the flexural tensile strength of mortars containing polypropylene fibres was more than 30% higher compared to fibre-free mortars, which confirmed the beneficial effect of dispersed reinforcement under room-temperature conditions. The analysis of results obtained at elevated temperatures showed that only at 300 °C a slightly lower flexural strength was recorded for fibre-reinforced mortars compared to reference mortars; however, this difference is not statistically significant. In the remaining cases, the addition of polypropylene fibres resulted in a slight increase in flexural tensile strength as compared to fibre-free mortars, reaching values of approximately 34% at 100 °C, which is statistically significant at the 95% confidence level (cf. Table 9 and Table 10), 10% at 200 °C (statistical significance below 80%), about 1% at 300 °C, and again approximately 10% at 400 °C, 500 °C, and 600 °C; these latter results are not statistically significant.

Table 9 was designed to present a comprehensive statistical analysis of flexural strength. When comparing means at a given statistical level, a three-step procedure needs to be applied, as described previously (see also [73]). First, it is necessary to verify whether the data within each group follow a normal distribution. The Shapiro–Wilk test was employed to assess whether the data followed a normal distribution. Where the assumption of normality was met, group comparisons were performed using either Student’s *t*-test or Welch’s *t*-test, depending on the homogeneity of variances, which was verified using Fisher’s test. If not, a non-parametric test (most commonly the Wilcoxon test) should be applied. The third column of Table 10 presents *p*-values for the Shapiro–Wilk test. If *p* > α, the null hypothesis of normality cannot be rejected. In all cases this condition is satisfied; however, at 500 °C the inference is sensitive to a slight change in α, and in such a case it is safer to use a non-parametric test. The fourth column presents the results of Fisher’s test comparing variances in both specimen sets: 0F and 1.8F mortars at a given temperature. This shows that at 100 °C and 600 °C the heterogeneity of variances is statistically significant; therefore, all comparisons should account for this assumption, and the most appropriate approach in such cases is the Welch’s *t*-test. This heterogeneity is also clearly visible in the data, which is why a separate analysis of variance differences was proposed in this study.

To assess the degree of homogeneity and repeatability of the obtained results, as well as to quantitatively describe the irregularity of the material degradation process as a function of temperature, the coefficients of variation (CV) of tensile strength were compared for the tested specimen series. In order to quantify the dispersion of the data, irrespective of the absolute values of the measurements, the coefficient of variation was used, defined as the ratio of the standard deviation to the mean. This metric is particularly appropriate for evaluating results obtained over a broad temperature range, where mean strength is subject to substantial reductions.

**Figure 14:**
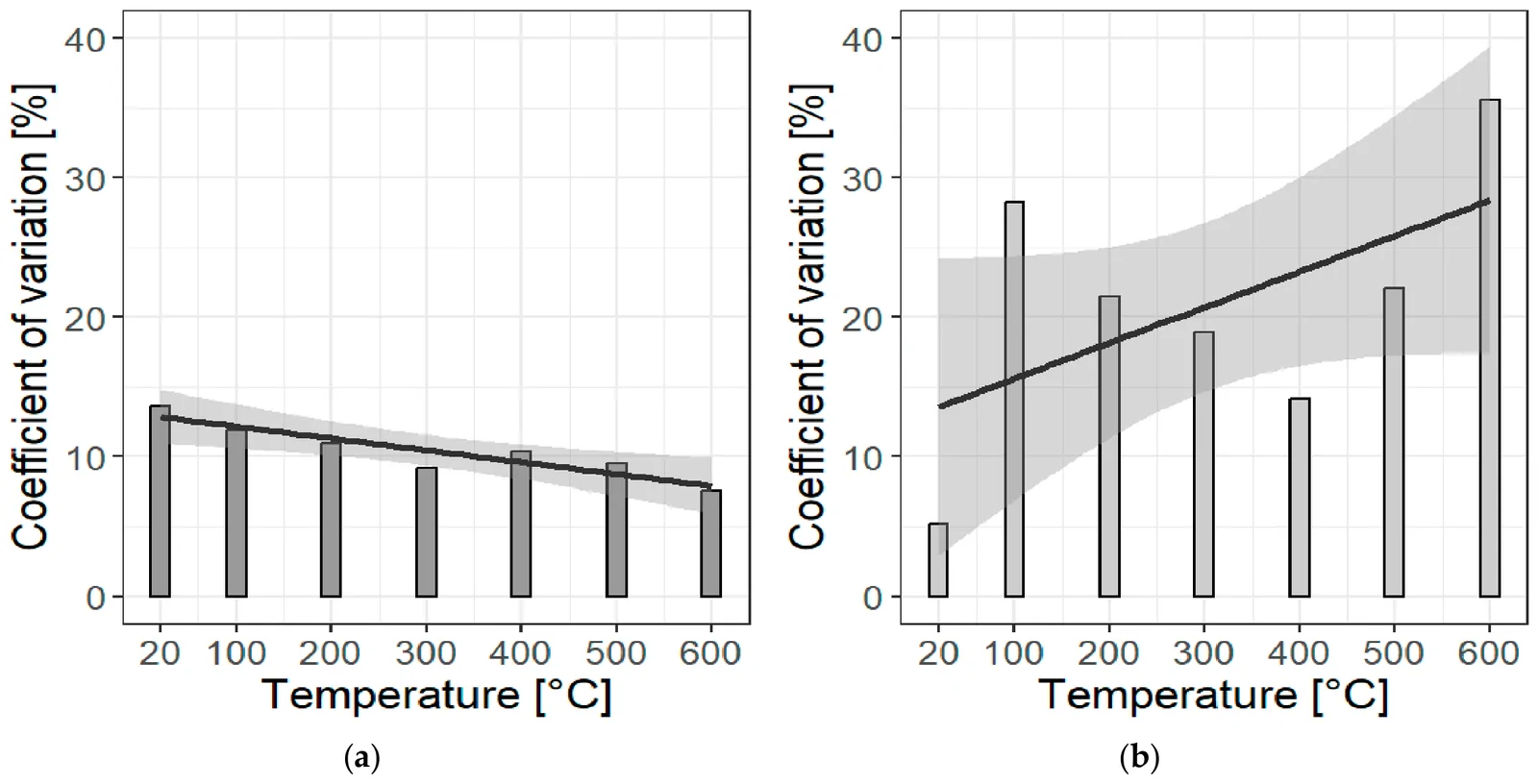
Calculated coefficient of variation as a function of temperature for mortar specimens without fibre addition (**a**) and polypropylene fibre-reinforced mortar specimens (**b**). In both plots, a linear fit with a confidence band was applied: 95% in (**a**) and 90% in (**b**) (the confidence level was reduced for aesthetic reasons, as the band significantly extended beyond the plot range).

For this reason, the comparison of coefficients of variation constitutes an important complement to the analysis of mean values and enables a more comprehensive assessment of the influence of temperature and fibre type on the mechanical behaviour of the tested material. The results of this analysis are presented graphically in Figure 14.

Figure 14 presents the relationship between the coefficient of variation (CV, %) and temperature for flexural testing of 4 × 4 × 16 cm prismatic specimens for both types of mixtures.

An analysis of the coefficient of variation (CV) of tensile strength results points to a significant increase in data scatter with increasing temperature, as observed in the experiment for PP fibre-modified mortar specimens. This phenomenon can be interpreted as the effect of the random distribution of channels formed after fibre melting, as well as local differences in the degree of cement matrix degradation. This effect is not observed for the reference specimens; on the contrary, the scatter was found to decrease slightly, although it is too small to draw statistically significant conclusions, and the hypothesis of constant CV cannot be rejected (Table 10).

The above table (Table 10) allows the conclusion that—provided the data in both groups (0 and 1.8 kg/m^3^) follow a normal distribution—the variances are significantly different (being clearly higher for the fibre-reinforced specimens). This requires the use of Welch’s parametric test for comparison. The result clearly indicates that the mean coefficient of variation for mortars with F-type fibre addition is higher (*p* < α, which implies the need of rejecting the null hypothesis in favour of the alternative hypothesis that assumes that the CV is greater in the fibre-reinforced group). Additionally, for PP fibre-reinforced mortars, the coefficient of variation exhibits a statistically significant increase with temperature. For fibre-free mortars, a slight decreasing trend is observed; however, the hypothesis of zero correlation cannot be rejected. Therefore, it can be assumed that the CV remains constant or shows a slight decrease with increasing temperature.

Another mechanical parameter analysed for the specimens considered optimal was the compressive strength. In contrast to tensile strength, this parameter is to a smaller extent governed by the initiation of individual cracks and more strongly reflects the overall state of densification and continuity of the cementitious matrix, as well as its ability to carry volumetric loads. Furthermore, compressive strength remains sensitive to microstructural changes induced by temperature, in particular dehydration of cement hydration products, the development of porosity, and degradation of interfacial transition zones (ITZ).

Two specimen types were used to determine compressive strength in the present study: 40 × 40 × 160 mm prism halves obtained after flexural testing, and 10 × 10 × 10 cm cubes used for determining the mortar strength class. This approach enabled the assessment of the influence of temperature, specimen geometry, and loading history on the obtained strength values.

**Figure 15:**
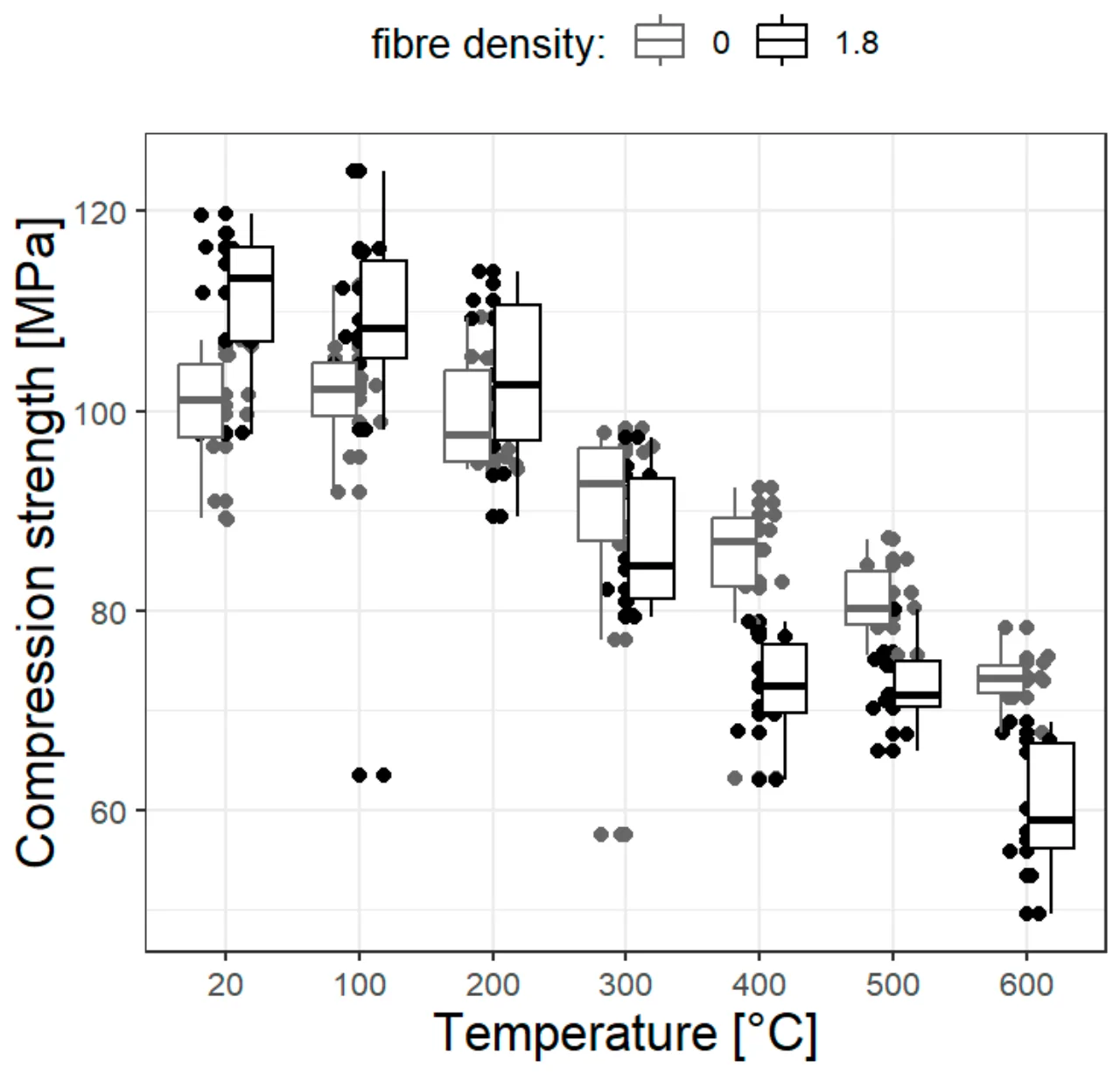
Point and box-plots of compressive strength results of mortar reference samples (grey) and samples reinforced with PP fibres (black).

Figure 15 presents the relationship between compressive strength and temperature for half-prism specimens (4 × 4 × 16 cm) made of reference mortars and mortars modified with F-type polypropylene fibres.

The compressive strength results demonstrated a consistent reduction with increasing temperature for both mortar types, following an almost linear trend. A slight increase in strength was observed only after exposure to 100 °C. For the unmodified mortar, the initial compressive strength of 99.9 MPa increased marginally to 105.8 MPa at 100 °C before progressively declining to 73.1 MPa at 600 °C. A similar trend was identified for the polypropylene-reinforced mortar, although its compressive strength decreased from 110.6 MPa under ambient conditions to 60.3 MPa after exposure to 600 °C. The effect of polypropylene fibres depended on the exposure temperature. Up to 200 °C, fibre incorporation had a favourable influence on compressive strength, with increases of approximately 15%, 10%, and 7% relative to the unreinforced mortar at 20 °C, 100 °C, and 200 °C, respectively. Above this temperature, with further increases in thermal exposure, a deterioration in compressive strength of the PP-reinforced composite was observed, with values lower by approximately 10% at 400 °C, 500 °C, and 600 °C.

**Figure 16:**
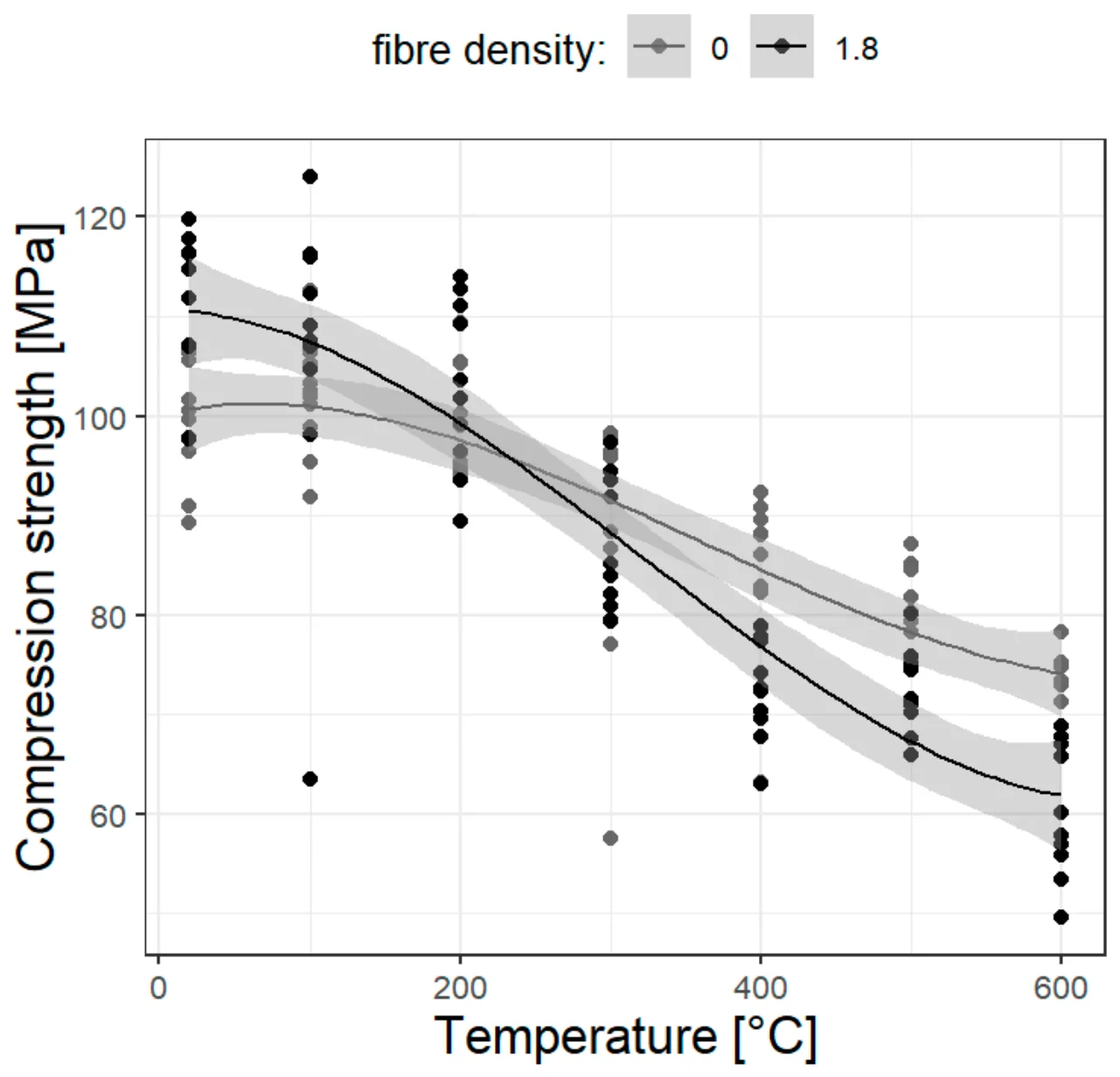
Relationship between compressive strength and temperature with a fitted third-degree polynomial for reference specimens (grey) and PP fibre-reinforced specimens (black). The polynomial fit better reflects the changes in strength up to approximately 300–400 °C compared to the linear function.

Interestingly, although the linear decrease is more pronounced in the case of mortar specimens with added F fibres at a dosage of 1.8 kg/m^3^ (see in Table 11 with an R^2^ value close to 0.80), a better fit is obtained in both cases using a third-degree polynomial, which indicates a plateau or a slower rate of decrease in the 100–400 °C temperature range (cf. Figure 16).

These results confirm the conclusion reported in [57], indicating that the degradation of mechanical properties (including tensile and compressive strength) is not linear. Instead, the system exhibits a certain inertia, manifested as a plateau in the temperature-dependent response, persisting up to approximately 300–400 °C. Only beyond this range does progressive degradation occur, leading to a significant reduction in both compressive and tensile strength.

To assess the degree of homogeneity and repeatability of the obtained compressive strength results, the coefficients of variation (CV) were again analysed for both types of specimens. The use of CV enables a quantitative description of data dispersion independently of their absolute level, which is particularly important in conditions of a significant reduction in mean strength values with increasing temperature.

The results indicate an increase in the mean coefficient of variation for fibre-reinforced (F) specimens as compared to plain cement mortar specimens; however, no correlation between the coefficient of variation (CV) and temperature was recorded in either group.

Table 12 presents a statistical analysis of the coefficient of variation calculated for groups of mortar specimens with and without fibre (F) addition at a given temperature. In the Shapiro–Wilk test, *p* > α (0.05), indicating that at the 95% confidence level the distribution of results does not differ significantly from a normal distribution. It can be assumed (Fisher’s test) that the variances in both groups are equal; therefore, the two groups can be compared using a parametric *t*-test, which provides more reliable results. From the fourth column of Table 12, it can be inferred that the coefficient of variation for the group of mortar specimens with fibre (F) addition is higher (at an 80% significance level; thus, the inference is relatively uncertain). However, there is no clear evidence as to whether increasing temperature leads to greater heterogeneity of results; the last column of Table 12 presents the *p*-value and the 95% confidence interval for the correlation. The *p*-value was calculated using Pearson’s correlation test. These results indicate that no correlation exists between the coefficient of variation and temperature in either group.

The next mechanical parameter analysed for the specimens considered optimal was the modulus of elasticity, which is a measure of material stiffness and its ability to elastically carry loads within the range of linear deformation. In contrast to strength, the modulus of elasticity is a parameter particularly sensitive to early microstructural changes, such as the formation of microcracks, the reorganisation of the C–S–H gel structure, and changes in the nature of interfacial interactions within the interfacial transition zones (ITZ). For this reason, its analysis provides important information on the degree of material degradation even before the failure state is reached.

In this study, the modulus of elasticity was determined on “dog-bone”-shaped specimens under tensile loading conditions. This approach enabled the assessment of the influence of temperature and the type of stress state on the elastic response of the material.

**Figure 17:**
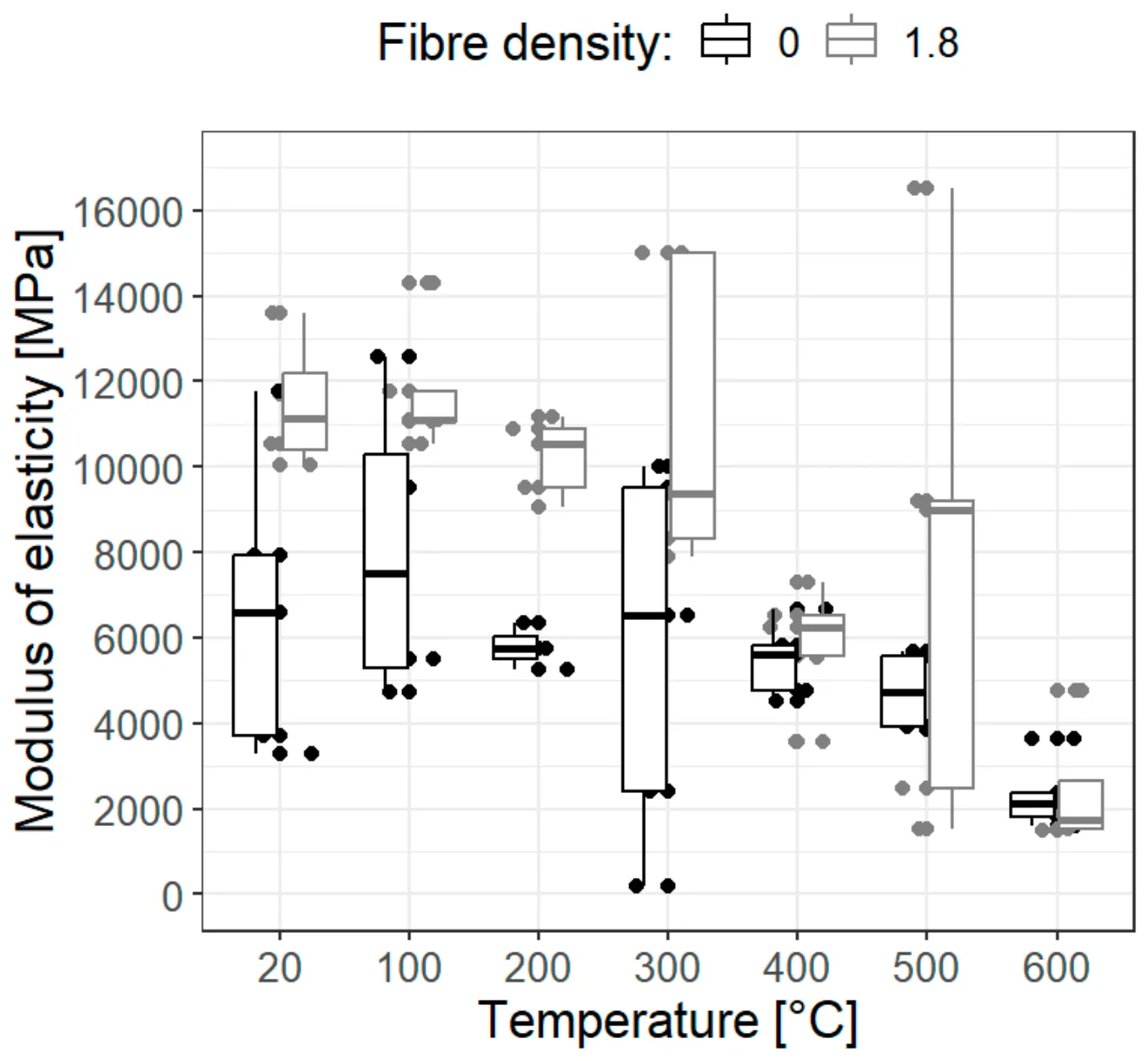
Graph of the modulus of elasticity [MPa] versus temperature in the group of mortar samples without fibre addition (black) and with PP fibre addition (grey). In addition to the scatter plot, a box-and-whisker plot was also used to represent descriptive statistics for both groups of mortars.

The effect of temperature on the modulus of elasticity of the dog-bone specimens is presented in Figure 17.

The results point to a progressive decline in the tensile modulus of elasticity with increasing exposure temperature for both unreinforced mortars and those containing polypropylene fibres. At 20 °C and 100 °C, the unreinforced specimens exhibited higher modulus values than the fibre-reinforced mortars. However, from 200 °C onwards, the rate of deterioration differed considerably between the two material types. Fibre-free mortars showed a reduction in the tensile modulus of elasticity of approximately 60%, whereas the corresponding decrease for polypropylene fibre-reinforced mortars was limited to about 45%. Consequently, throughout the temperature range from 200 °C to 600 °C, the mortars incorporating polypropylene fibres retained higher tensile modulus values than the unreinforced specimens. At 600 °C, both mortar groups exhibited a comparably high degree of degradation of the modulus of elasticity, amounting to approximately 65% for fibre-free mortars and approximately 79% for fibre-reinforced mortars.

To assess the homogeneity and repeatability of the obtained results, the coefficient of variation (CV) of the modulus of elasticity was once again analysed for both types of specimens. The use of the CV enables a quantitative description of data dispersion independent of their absolute level, which is particularly important under conditions of a significant reduction in the modulus values with increasing temperature.

**Figure 18:**
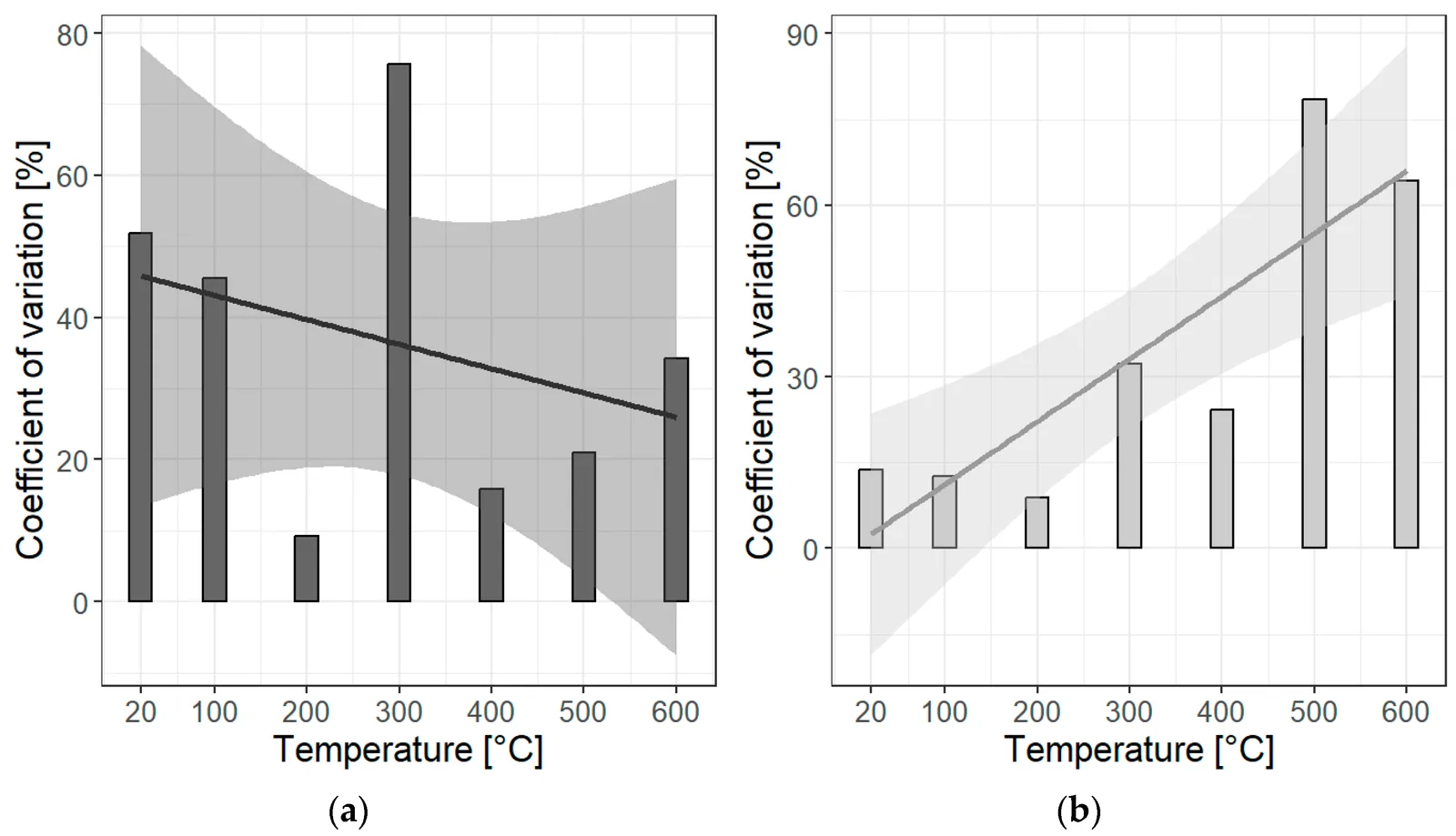
Coefficient of variation as a function of temperature for mortars without fibre addition (**a**) and with PP fibre addition (**b**). A regression analysis together with a 95% confidence interval was also performed. The variability of the CV is not evident in the case of the reference specimens (wide confidence interval); however, a clear increase in CV with temperature can be observed for the mortar specimens reinforced with PP fibres.

Results of the coefficient of variation analysis are presented in Figure 18 as a function of temperature for reference mortars (0F) and fibre-modified mortars (1.8F) obtained in tensile modulus of elasticity tests using the axial tension method.

An analysis of the coefficient of variation (CV) for the modulus of elasticity indicates a distinct increase in the scatter of results with increasing temperature, particularly for specimens subjected to tensile testing. This phenomenon may be interpreted as a consequence of growing microstructural heterogeneity, local differences in the degree of cement matrix degradation, and the stochastic nature of microcrack initiation.

These conclusions are supported by the statistical analysis performed for the mortars reinforced with F fibres; however, in the case of the reference mortars, the interpretation is associated with considerable uncertainty and should therefore be approached with caution (Table 13).

In mortars containing PP fibres, a tendency may be observed towards limiting the increase in CV within the intermediate temperature range, indicating more stable and reproducible transfer of elastic strains.

The findings of the present study are consistent with previously published research on the behaviour of cement-based materials exposed to elevated temperatures. While the majority of available studies have focused on concrete containing coarse aggregate rather than cement mortars, they provide a useful basis for comparison. Hertz [39], for example, proposed idealised strength–temperature relationships for concrete, identifying the general pattern of mechanical performance during thermal exposure. These relationships suggest that concrete may exhibit a slight increase in strength within the lower temperature range (approximately 100–200 °C), which is universally attributed to the evaporation of free water and physicochemical changes that take place within the cement paste. As the exposure temperature increases further, this initial improvement is followed by a pronounced decline in strength. The extent and rate of deterioration are governed by several factors, including the concrete strength class, the type and volume fraction of aggregate, moisture content, heating rate, and the loading conditions applied during thermal exposure. Given their broad applicability, the strength–temperature relationships proposed by Hertz have become a widely accepted reference for both fire engineering design and comparative studies reported in the literature. The primary comparative observation indicates that cement mortars exhibit a trend similar to that described by Hertz’s curves—namely, an initial increase in strength up to approximately 100–200 °C, followed by a subsequent decline. In contrast, the reduction in compressive strength observed in the present study was less pronounced than that typically reported for concretes containing coarse aggregate, where strength losses of 40–70% relative to room-temperature values are commonly recorded after exposure to 500–600 °C. This behaviour is most likely associated with the more homogeneous microstructure of cement mortar that consists solely of a cementitious matrix and fine aggregate. Unlike conventional concrete, mortar does not contain coarse aggregate, thereby minimising thermal incompatibility between its constituents. As a result, a reduction takes place in the development of internal stresses and thermally induced microcracking, contributing to a slower deterioration of load-bearing capacity at elevated temperatures. Furthermore, smaller dimensions of the mortar specimens (smaller cross-sections) promote more uniform heating and reduce temperature gradients, which in turn minimises local thermal stresses. In contrast, larger concrete elements are characterised by more pronounced temperature gradients, leading to increased microcracking and a more rapid loss of strength. The initial increase in strength observed at approximately 100 °C may be associated not only with evaporation of free water, but also with partial “condensation” and recrystallisation of hydration products within the cement matrix, as well as additional energy-release processes related to the continued hydration of C–S–H phases.

The findings of the present study are also consistent with recent reports describing the effects of elevated temperatures on the mechanical properties of mortars and concretes [51,52,53,54,55]. An analysis of the mean flexural and tensile strengths revealed a characteristic non-linear pattern of variation: an increase in values up to approximately 100 °C, a decrease at 200 °C, an atypical local increase at 300 °C, followed by a further decline at higher temperatures.

The initial increase in strength within the low-temperature range (up to approximately 100–200 °C) can be justified by chemical reactions occurring within the cement matrix and energy dissipation processes. The evaporation of free water and partially bound water from the capillary pores.

H_2_O_(l) + H_2_O_(c) → vapour (10)

This leads to structural drying, which increases the local density within the C–S–H phase (xCaO·SiO_2_·yH_2_O) and limits plastic deformations. Moreover, mechanical energy associated with deformation is partially absorbed through microstructural reorganisation of crystallites and rehydration of incompletely bound hydration products.

C_3_S + 3H_2_O → C–S–H + Ca(OH)_2_ (11)

C_2_S + 2H_2_O → C–S–H + Ca(OH)_2_ (12)

CaSO_4_·½H_2_O + C_3_A + 2H_2_O → ettringite Ca_6_Al_2_(SO_4_)_3_(OH)_12_·26H_2_O) (13)

In particular, further hydration of cement within the temperature range up to 200 °C (reactions of C_3_S and C_2_S, as indicated above, as well as partial dehydroxylation of portlandite:

Ca(OH)_2_ → CaO + H_2_O (14)

at the upper limit of the temperature range) enhances the cohesion of the matrix and leads to a temporary increase in strength. These processes simultaneously dissipate energy, as crystal reorganisation and partial rehydration consume energy associated with thermo-mechanical deformation.

A similar mechanism was observed in studies on AAS (alkali-activated slag) mortars by Le et al. [52], where an increase in compressive strength of 36.9% was reported at 200 °C. The authors attributed this effect to ongoing hydration processes (SiO_2_ + NaOH/KOH + H_2_O → C–S–H gel) and microstructural densification, which may also explain the local increase in strength observed in the present specimens at 300 °C. This behaviour can be attributed to partial reconsolidation of the cement paste following the release of water vapour from the pore structure and the gradual recrystallisation of C–S–H products (xCaO·SiO_2_·yH_2_O → new C–S–H), which further dissipate stress energy within the microstructure.

Regarding the effect of polypropylene (PP) fibres on mechanical performance, the findings obtained in this study correspond closely with trends reported in previous research. For specimens tested without thermal exposure, fibre incorporation led to a 20% increase in tensile strength compared with the unreinforced mortar. Similar improvements were maintained after heating, with strength increases of approximately 25% at 100 °C, 10% at 200 °C, and ca. 1% at both 400 °C and 600 °C. These observations are compliant with results presented by Ezziane et al. [53], who showed that polypropylene fibres contribute not only to higher compressive and flexural strength but also to increased fracture energy and improved deformability of cement-based materials. This effect was associated with the limitation of microcrack development and a more uniform redistribution of stresses within the material. In the present study, PP fibres exhibited a comparable reinforcing mechanism, enhancing tensile resistance—particularly at lower temperatures—before their effectiveness was reduced due to thermal degradation. (at ca. 160–170 °C). The melting of fibres and the partial vapour-related dissipation of their volume within the capillary network leads to the formation of microchannels for water vapour transport, which reduces internal stresses in the matrix and limits the formation of sudden microcracks. In this sense, the fibres act as elements that dissipate thermo-mechanical energy within the composite structure.

This phenomenon can also be directly related to the findings of Han et al. [54] who reported a porosity level of 5.89% at 400 °C in ultra-high-performance concrete (UHPC) reinforced with polyethylene fibres (PEFs), as well as the possible reduction in explosive spalling, while maintaining a compressive strength of 98.3 MPa at 400 °C and 36.0 MPa at 800 °C. Although polypropylene fibres differ from PEFs in terms of their thermal characteristics, particularly with respect to melting temperature (approximately 160–170 °C for PP fibres compared with 260–270 °C for PEFs), the relatively limited decline in flexural and tensile strength observed in the present study above 400 °C may be attributed to a comparable mechanism. This mechanism involves the development of controlled micro-porosity, which limits the risk of rapid increases in vapour pressure and allows partial dissipation of mechanical energy through deformation of the internal microstructure. This process can be presented schematically as follows: PP (melting) → partial evaporation → microchannels → reduction in vapour pressure → lower internal stresses → limited degradation of mechanical strength. During fibre melting, an endothermic heat absorption occurs (ΔH melting), which additionally dissipates thermal energy and mechanically counteracts stress concentrations in pore-adjacent zones. Furthermore, local recrystallisation of residual polymer within the channels may act as a “viscous inclusion”, absorbing part of the mechanical energy generated during water vapour expansion.

The observations of Kong et al. [55] for UHPC incorporating hybrid steel–polypropylene fibre reinforcement provide further support for the positive role of polypropylene fibres in maintaining the mechanical performance of cement-based materials at elevated temperatures. Their results showed that up to approximately 400 °C, the material exhibited a slight strength increase of several percent, and subsequently a progressive deterioration as the exposure temperature increased even further. The authors attributed this behaviour to the preservation of a higher level of structural integrity under rapid heating conditions, which may also explain the relatively limited differences in mechanical properties recorded in the present study at 400 °C and 600 °C.

From the perspective of chemistry and energy, this mechanism involves fibre melting (PP → ΔH of fusion, energy absorption), partial decomposition/vaporisation of the polymer (PP → gaseous phase + residual polymer, thermal energy dissipation), formation of microchannels (reduced vapour pressure → lower internal stresses → reduced microcracking in the C–S–H matrix), and local recrystallisation of residual polymer (additional absorption of mechanical energy and further dissipation). Consequently, PP fibres contribute to the development of controlled micro-porosity and the mitigation of abrupt failure processes, thereby maintaining relatively stable mechanical properties of concrete up to temperatures of approximately 400 °C.

Overall, the results acquired in this study were found to be consistent with the initial improvement in mechanical properties of cement-based mortars and concretes commonly reported in the literature for the 100–200 °C temperature range [51,52]. The local deviations observed at approximately 300 °C may be associated with moisture evaporation (H_2_O(l) → H_2_O(g)) and temporary changes in the internal microstructure, leading to partial reconsolidation of the material (partial rehydration of C–S–H products and recrystallisation of Ca(OH)_2_). These processes lead to the dissipation of mechanical energy during deformation.

Additionally, the observed beneficial influence of polypropylene (PP) fibres on tensile and flexural strength is consistent with studies on UHPC reinforced with polymeric and steel fibres [53,54,55]. The mechanism of fibre action involves heat absorption during melting (PP → ΔH of fusion), partial volatilisation of the fibres, and the formation of microchannels [75,76,77] that reduce local water vapour pressure within the concrete pores. This, in turn, limits microcracking and dissipates mechanical energy generated by thermal stresses.

Although numerous studies have addressed the behaviour of fibre-reinforced cement-based materials at elevated temperatures, the present findings provide additional evidence that mortars incorporating PP fibres can retain favourable mechanical performance even after exposure to temperatures as high as 600 °C. While the reinforcing effect of polypropylene fibres diminishes once their melting point is exceeded, their presence continues to contribute an improvement in flexural and tensile resistance compared with unreinforced specimens. This beneficial effect can be attributed to the formation of controlled microchannels during fibre degradation, which facilitates energy dissipation and contributes to the mitigation of thermal and mechanical damage.

The morphology of fracture surfaces of cementitious composite specimens containing polypropylene fibres was examined using a Nova NanoSEM 230 scanning electron microscope from FEI, equipped with a field emission gun (FEG). SEM images were recorded with a low-vacuum secondary electron detector (LVD). Image acquisition was performed under low-vacuum conditions according to the following parameters: accelerating voltage of 5–10 kV, chamber pressure of 0.6 Torr in a water vapour environment, and magnification ranging from 150× to 2400×. Specimens intended for analysis were mounted on microscope stubs using double-sided conductive carbon adhesive tape. The composite samples were not coated with a conductive layer prior to examination.

**Figure 19:**
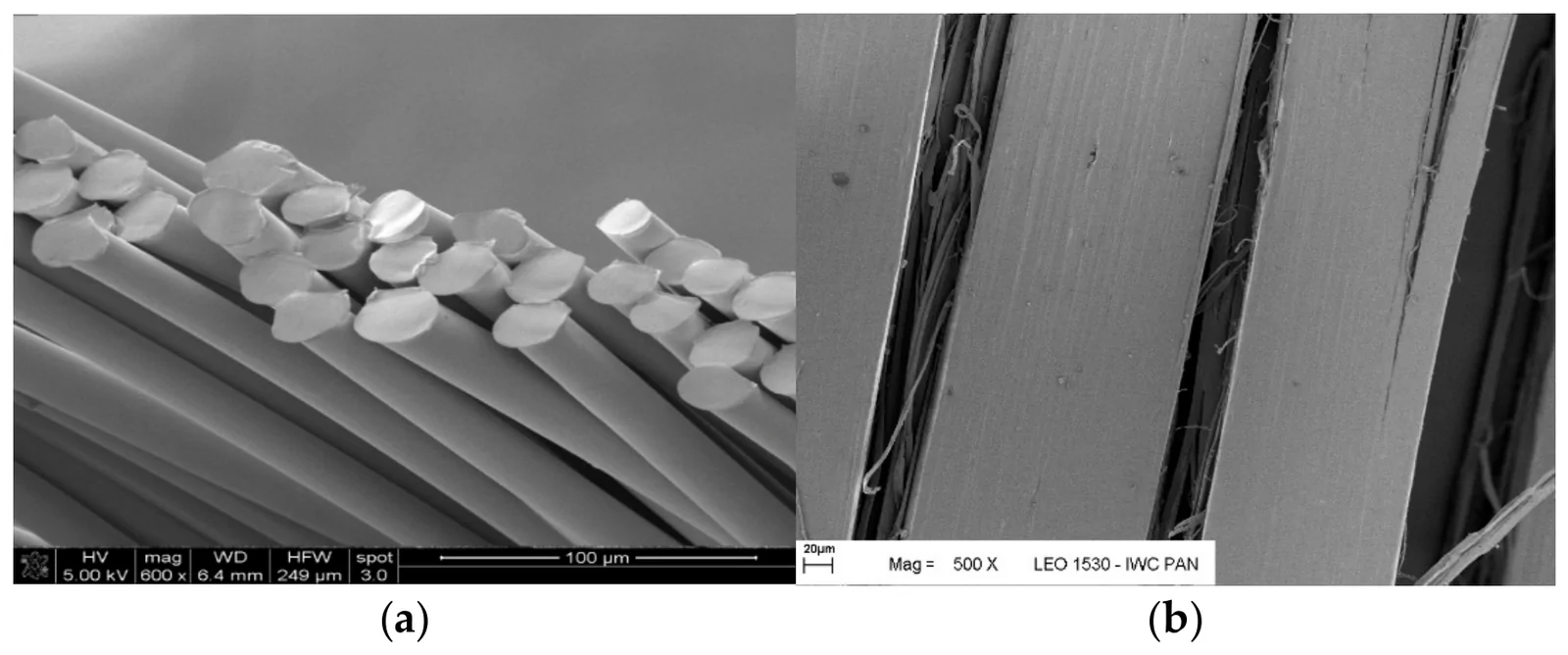
SEM of polypropylene fibres: (**a**) I—Ignis; (**b**) F—Fibrofor Fibre High Grade 190.

SEM images of polypropylene fibres designated as “I” (Figure 19a), obtained at a magnification of 600×, enabled precise establishing of their cross-sectional geometry and estimation of fibre diameter. The fibres were produced through a conventional polymer melt-spinning process. They are characterised by a regular, circular cross-section and a smooth surface, without any distinct morphological surface features. The fibre diameter was approximately 20 µm.

SEM images of polypropylene fibres designated as “F” (Figure 19b) were obtained at magnifications ranging from 150× to 600×. The micrographs clearly indicate that the fibres were produced by a film fibrillation process, which involves controlled mechanical splitting of a thin polymer film into a network of interconnected microfibres. As a result, a material with an increased specific surface area and a highly developed spatial structure is obtained, and that promotes effective anchorage within the cementitious matrix. The use of such fibrillated polymer fibres as dispersed reinforcement in concrete contributes to the reduction in shrinkage-induced cracking during the curing stage, improves resistance to microcrack propagation, and increases the ductility of the composite. A further advantage is the uniform dispersion of fibres throughout the concrete volume with only a minor effect on the workability of the mix. However, as compared with the homogeneous type I fibres, the SEM images of type F fibres reveal a pronounced variability in cross-sectional dimensions. A significant proportion of fibres exhibit a rectangular cross-section of approximately 100 µm × 400 µm, while numerous much thinner fibrillar–fibrous fragments with irregular cross-sectional shapes and a thickness of (2–3) µm are also observed.

**Figure 20:**
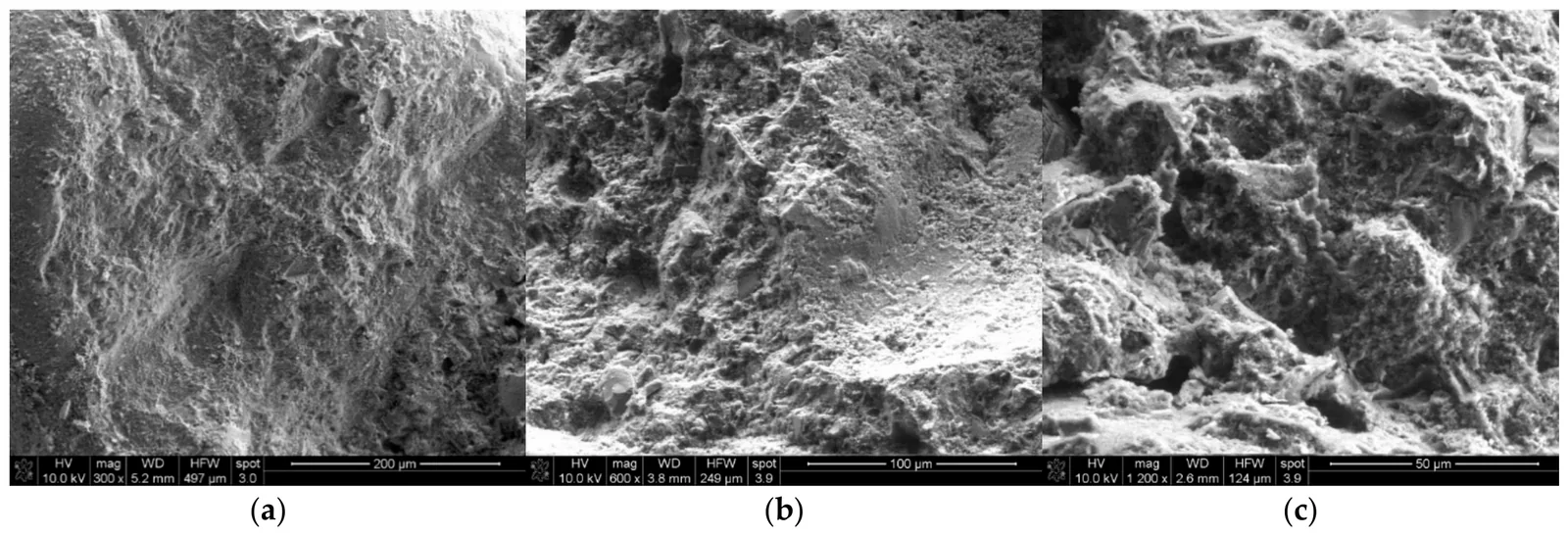
SEM images of fracture surfaces of fibre-free specimens: (**a**) 20 °C; (**b**) 200 °C; (**c**) 500 °C.

SEM images of fracture surfaces of cement mortar specimens without polypropylene fibres reveal significant changes in the nature of the failure surface as a function of exposure temperature. In the reference state (20 °C), the fracture surface is relatively smooth and homogeneous (Figure 20a), indicating a quasi-ductile micro-scale failure mechanism associated with the rupture of a continuous, still well-bonded cement matrix. In those conditions, the fracture energy is to a large extent absorbed by deformation of the C–S–H gel and the gradual propagation of microcracks within the hydration products.

With increasing thermal exposure temperature, a gradual increase in the development and “roughness” of the fracture surface is observed (Figure 20b,c). This should not be directly interpreted as an increase in the intrinsic porosity of the material, but rather as an intensification of topographical irregularities caused by a change in the failure mechanism. In the temperature range up to approximately 200–300 °C, the dominant phenomenon is dehydration of physically bound water from the C–S–H gel, leading to its shrinkage, weakening of interfacial bonds, and an increase in internal stress concentrations. This process leads to a reduced ability of the matrix to dissipate energy, while cracking becomes increasingly localised and abrupt in nature.

At higher temperatures exceeding 300 °C, an intensification of C–S–H gel dehydration and portlandite dehydroxylation occurs, causing a breakdown of hydration bonds and a reorganisation of the silicate structure. At the microstructural level, this process causes a transition from chemically hydrated bonding, characteristic of low-temperature phases, to more rigid but concurrently brittle ionic–covalent bonding typical of ceramic-like phases. Such a change in the nature of bonding increases the local stiffness of the matrix, while at the same time reducing its ability to relax stresses and dissipate strain energy.

Further temperature increases (above 300–400 °C) result in progressive degradation of the C–S–H gel structure, including partial depolymerisation of its silicate network and decomposition of portlandite Ca(OH)_2_, accompanied by a release of water vapour. These phenomena lead to a significant reduction in microstructural cohesion and a further increase in material brittleness, which is reflected in a highly developed, irregular fracture surface characteristic of brittle failure. In this temperature range, fracture energy is no longer effectively absorbed by microstructural deformation but instead leads to an abrupt rupture of weakened regions of the matrix.

**Figure 21:**
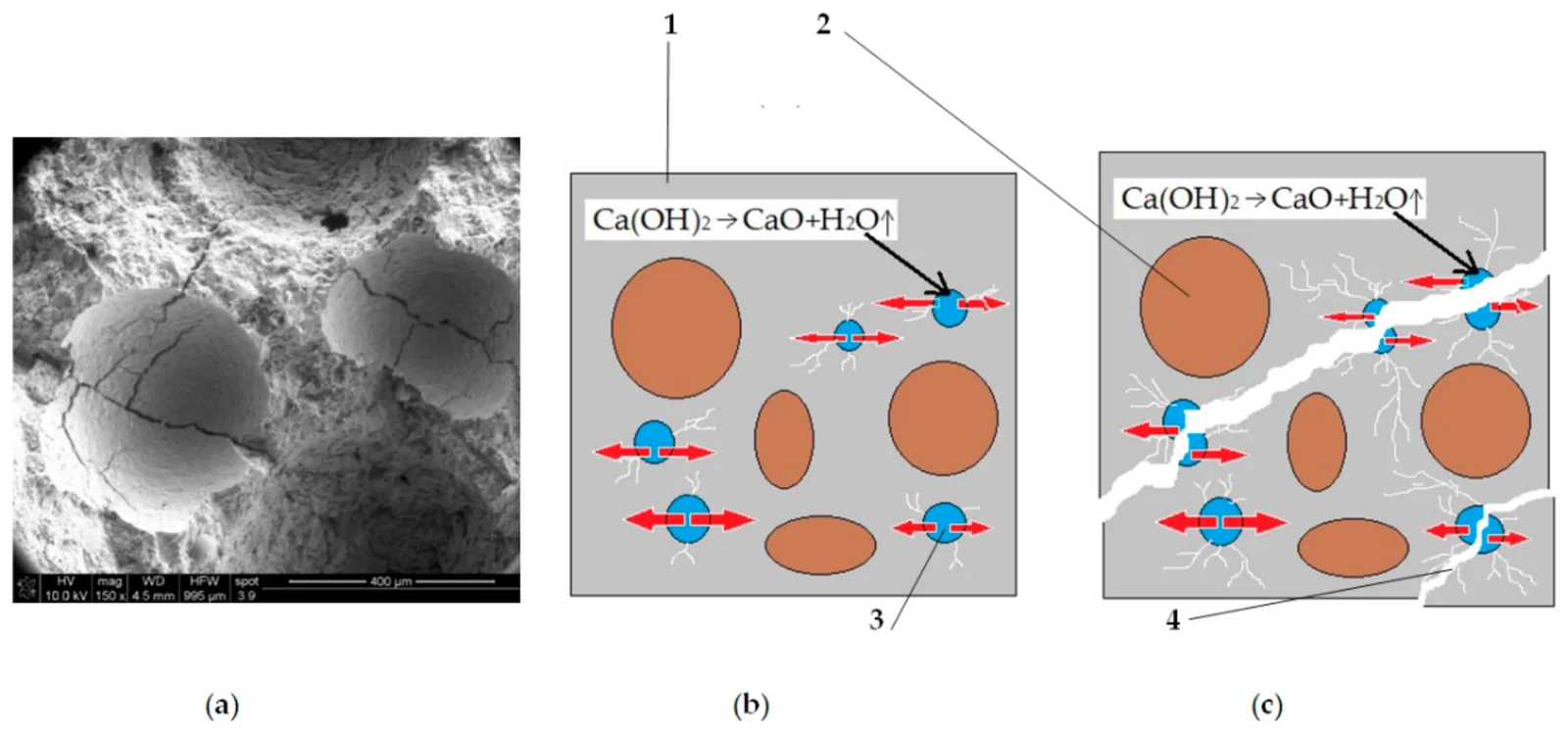
SEM image and schematic representation of microcrack formation on the pore walls of cement mortar at 600 °C: (**a**) SEM image showing visible cracks; (**b**) schematic of the composite subjected to heating; (**c**) schematic of thermal stress development within the pores of the composite; 1—cement paste; 2—aggregate; 3—pores containing capillary water; 4—crack propagation leading to structural failure of the composite and spalling phenomenon.

At the highest analysed temperature (600 °C), SEM images additionally reveal local microcracks appearing within existing pores or structurally weakened zones. Their presence can be associated with a sudden increase in water vapour pressure within closed pore spaces, as well as with the intensification of thermal stresses arising from differential thermal expansion of mineral phases. These phenomena lead to the initiation and propagation of microcracks, which constitute direct evidence of advanced microstructural degradation of the cement mortar at exposure to high temperatures. The SEM image and a schematic representation of microcrack formation on the pore walls of the cement mortar are presented in Figure 21.

**Figure 22:**
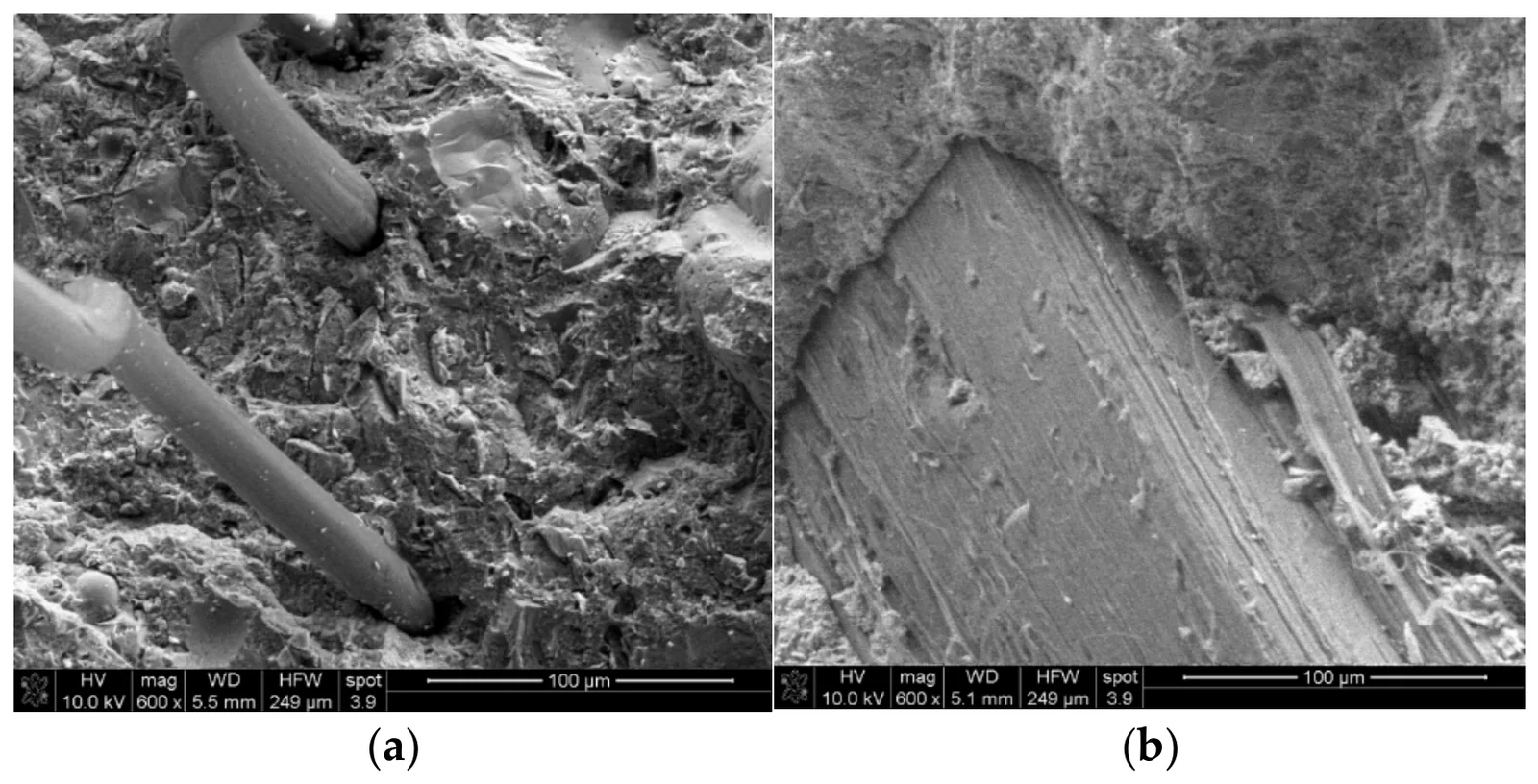
Images of fibre-reinforced composites at temperatures below the fibre decomposition temperature: (**a**) I—Ignis, 20 °C; (**b**) F—Fibrofor Fibre High Grade 190, 100 °C.

The absence of microcrack-bridging mechanisms in fibre-free specimens promotes rapid crack development under mechanical loading. From the energy-based perspective, a material without fibres is characterised by a limited capacity to absorb and dissipate external energy, which causes low resistance to thermo-mechanical impact and results in a sudden, poorly controlled failure mode under elevated temperatures or after thermal exposure. On the fracture surfaces of concrete specimens that contain polypropylene fibres not subjected to thermal treatment (Figure 22), as well as those heated to 100 °C, fibres were observed to retain their original geometry corresponding to the form used in composite production. The absence of visible deformation, partial melting or morphological changes indicates that neither the concrete-mixing process nor thermal exposure up to 100 °C leads to physical or chemical degradation of PP fibres, whose softening and melting temperatures are significantly higher than the analysed range. Moreover, this stability confirms the chemical inertness of polypropylene in relation to the alkaline environment of both fresh and hardened concrete in the tested conditions.

An analysis of the fracture surface characteristics suggests that the failure process of cementitious composites containing PP fibres proceeded via two coexisting mechanisms: fibre rupture and partial fibre pull-out from the surrounding cement matrix. The presence on the fracture surfaces of protruding fibre segments with lengths ranging from approximately 200 µm to several millimetres provides clear evidence of an active pull-out mechanism, which plays a key role in enhancing the material’s ability to absorb fracture energy.

Regarding the fracture energy balance, the fibre pull-out mechanism leads to a significant increase in the energy dissipated during crack propagation. This energy is consumed in overcoming friction at the fibre–matrix interface, inducing local deformation of the C–S–H gel, and progressively weakening the mechanical adhesion between the fibre and the cement hydration products. In contrast to the brittle failure observed in unreinforced mortars, the presence of fibres introduces a crack-bridging mechanism that delays crack growth and promotes a more distributed mode of fracture.

In the analysed temperature range (up to 100 °C), chemical interactions at the fibre–matrix interface remain unchanged, and fibre–matrix bonding is predominantly mechanical in nature, which is a result of fibre anchorage within the C–S–H gel structure and the micro-roughness of the fibre surface. The absence of any signs of fibre degradation or loss of continuity indicates that the fibres’ ability to transfer tensile stresses and effectively dissipate fracture energy is preserved. Consequently, the composite exhibits increased resistance to crack initiation and propagation compared with unreinforced concrete.

**Figure 23:**
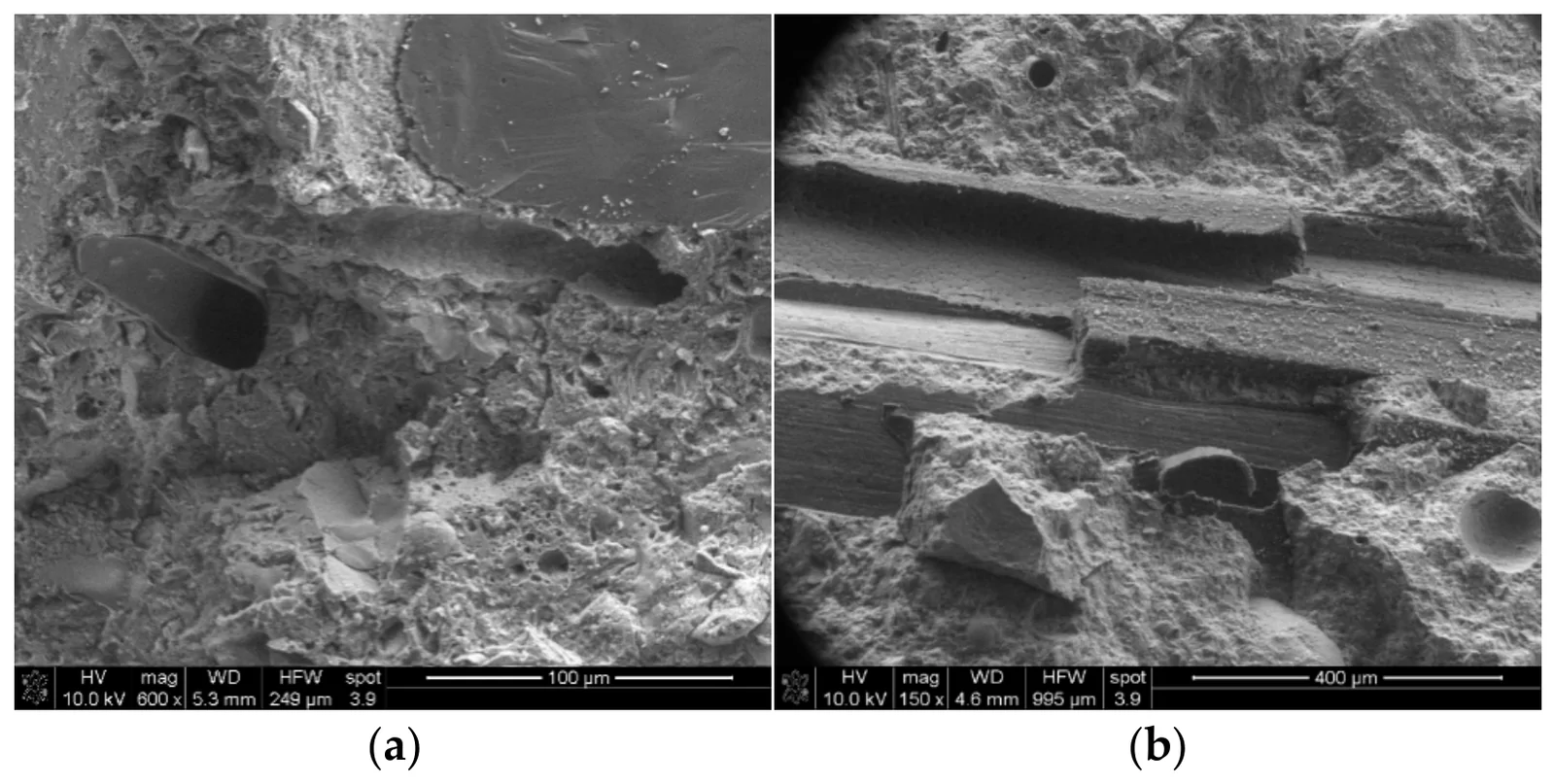
Images of fibre-reinforced composites at the temperature initiating fibre degradation: (**a**) I—Ignis, 200 °C; (**b**) F—Fibrofor Fibre High Grade 190, 200 °C. In both images, remnants of fibres deformed under thermal exposure are visible.

Thermal treatment of cementitious composite specimens containing polypropylene fibres at 200 °C clearly exceeds the melting temperature of PP fibres, leading to a fundamental change in their role within the material structure (Figure 23). During the 30 min thermal exposure, the fibres lose their original geometric form and their ability to mechanically bridge cracks, transitioning into a low-viscosity molten state. The molten polymer, driven by surface energy gradients and capillary phenomena, penetrates the surrounding porous cement matrix, particularly into regions associated with the C–S–H gel and microvoids formed as a result of partial dehydration of cement hydration products.

Once the specimens have been subjected to thermal exposure and then cooled, only a small amount of re-solidified polymer is observed to remain within the channels corresponding to the original fibre locations. In the SEM images of F/200 °C specimens, a thin polymer layer with a thickness of approximately 10–15 µm can be seen adhering to the channel walls with a rectangular cross-section (approximately 100 µm × 400 µm), which was previously completely filled by the fibre. This phenomenon indicates a substantial outflow of molten polypropylene into the surrounding concrete microstructure during heating, and consequently the formation of a permanent void in the form of a fibre-derived macrochannel.

Regarding thin PP fibres with a circular cross-section, after melting and subsequent solidification the polymer adopts the form of thin-walled tubes adhering to the walls of channels previously occupied by the fibres. This morphology of the re-solidified polymer comes from a combination of molten polymer flow, its adhesion to the mineral components of the matrix, and volumetric shrinkage during cooling. The observed morphology indicates that most of the fibre volume has been effectively transferred to the porous structure of the concrete, while the polymer itself remains solely as a thin coating deposited on the internal surfaces of the resulting channels.

From an energy and mechanical viewpoint, a temperature of 200 °C marks a transition point between conventional dispersed reinforcement and a mechanism of indirect fibre influence on composite behaviour. The energy that was dissipated at lower temperatures through fibre rupture and pull-out is instead expended on the processes of melting, flow and redistribution of the polymer within the concrete microstructure. Moreover, the resulting fibre channels play an important role in resistance of the material to elevated temperatures by enabling the release of water vapour generated by C–S–H gel dehydration and portlandite decomposition. As a result, the risk of water vapour pressure build-up and subsequent explosive cracking of the concrete structure at higher temperatures is reduced.

Exposure of cementitious composite specimens to higher temperatures (300 °C, 400 °C, 500 °C, and 600 °C) leads not only to complete melting of polypropylene fibres but also to a fundamental change in rheological properties of the molten polymer. With increasing temperature, a significant reduction in the viscosity of molten PP occurs, markedly enhancing its mobility and ability to penetrate the porous and microcracked cement matrix. Under such conditions, the molten polymer readily migrates into the capillary pore system, interfacial microcracks, and microdefects formed as a result of progressive dehydration of cement hydration products.

**Figure 24:**
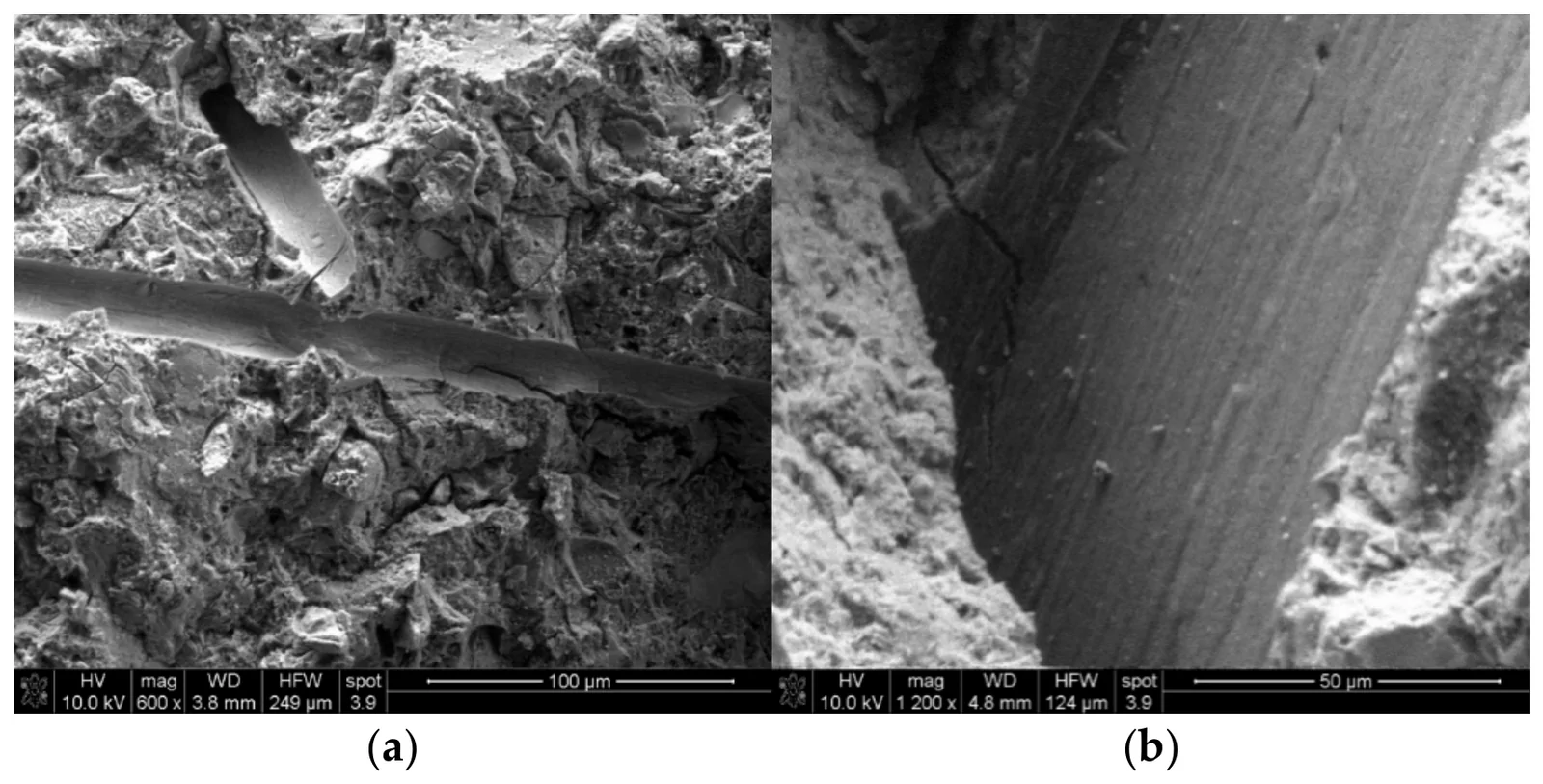
Images of fibre-reinforced composites at temperatures above the fibre decomposition temperature: (**a**) I—Ignis, 500 °C; (**b**) F—Fibrofor Fibre High Grade 190, 500 °C. In both images, empty channels formed after fibre melting are visible.

Consequently, after completion of the thermal exposure and subsequent cooling of the specimens, only trace amounts of solidified polymer remain at the original locations of the PP fibres, significantly less than in specimens heated to 200 °C. The fibre channels are almost completely emptied (Figure 24), forming open, continuous voids of a capillary–crack-like nature. This phenomenon is confirmed by SEM images of the fracture surfaces, where solidified residues of melted PP fibres are practically indistinguishable or appear only locally in the form of thin, discontinuous polymer films.

**Figure 25:**
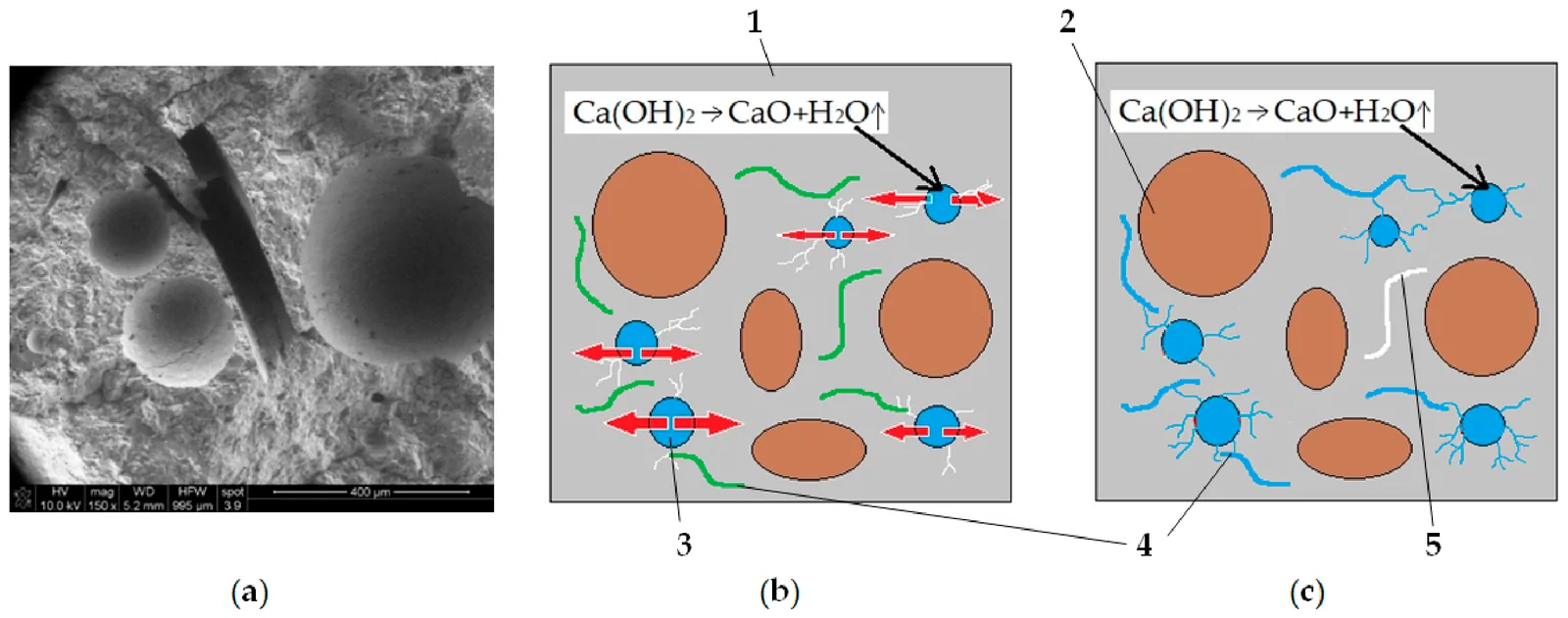
SEM image and schematic representation of the reduction in microcrack formation on the pore walls of the cementitious composite induced by polypropylene fibres at 300 °C: (**a**) pore structure with significantly reduced cracking in the vicinity of the void formed after melting of type F fibre; (**b**) diagram of the composite during thermal exposure; (**c**) schematic of thermal stress energy dissipation within the composite pores through channels formed after fibre melting; 1—cement stone, 2—aggregate, 3—pores containing capillary water, 4—fibres and schematic representation of water vapour dispersion and thermal energy dissipation through channels formed by the disappearance of polypropylene fibres, 5—channel formed as a result of fibre melting.

From the energy-based perspective, the observed process indicates that the energy supplied to the system at high temperatures is predominantly consumed by the intensified mass transport of molten polymer and the ongoing reconfiguration of the cement matrix microstructure, rather than by mechanical interaction between fibres and cracks. What is more, the near-complete removal of polymer from the fibre channels promotes the formation of an effective network of gas migration pathways, which is of particular importance under continued thermal exposure. The resulting channels facilitate the release of water vapour generated by C–S–H gel dehydration and the decomposition of portlandite Ca(OH)_2_, limiting internal pressure build-up and reducing the risk of sudden structural degradation of the composite. As a result, in the temperature range above 300 °C, the role of polypropylene fibres undergoes a radical transition: from active dispersed reinforcement at low and moderate temperatures to a structural component whose melting and migration lead to a beneficial reorganisation of the cement composite microstructure under high-temperature conditions. The SEM image and schematic representation of the mitigation of microcrack formation on the pore walls of cement mortar through the use of polypropylene fibres are presented in Figure 25.

The results of laboratory testing will be used to evaluate reliability models developed according to an innovative research program. Specialised software is planned for deployment of reliability modelling (probability of failure/non-failure). The test results will enable the development of empirical and theoretical distributions, estimation of probability of failure/non-failure, and graphical analysis and additional analyses [78,79].

## 4. Conclusions

The experimental study of high-strength, fine-grained cement mortars reinforced with polypropylene fibres under simulated fire exposure conditions allowed for the identification of key mechanisms responsible for changes in mechanical performance and microstructural degradation of the material.

The optimisation studies, which comprise the assessment of tensile strength for various types of fibre, clearly demonstrated the beneficial effect of dispersed reinforcement on the thermal resistance of cement mortars. Among the investigated fibre types, Fibrofor fibres were found to have the highest effectiveness throughout the entire temperature range, contributing to greater initial strength and a more stable mechanical response under thermal exposure. Although Ignis fibres also improved the mechanical performance of the composite, their influence was less consistent, particularly at approximately 100 °C, where a temporary decrease in strength compared with the reference specimens was observed. Regarding tensile strength, determined by flexural testing and direct tension tests on “dog-bone” specimens, both unreinforced mortars and fibre-modified mortars exhibited an increase in this parameter up to approximately 100 °C (from 8.3 MPa to 9.6 MPa for mortars without fibres and from 10.6 MPa to 12.5 MPa for fibre-reinforced mortars). This phenomenon may be associated with energy balance of the heating process, in which thermal energy leads to drying of the microstructure, moisture redistribution, and local densification of the cement matrix. At higher temperatures, the tensile strength followed an almost linear decreasing trend, reaching 5.1 MPa for the unreinforced mortar and 5.5 MPa for the fibre-reinforced specimens at 600 °C. Nevertheless, polypropylene fibres provided a measurable improvement in tensile performance throughout the entire temperature range investigated.

The assessment of compressive strength demonstrated a progressive, nearly linear deterioration with an increase in temperature. The strength decreased from 99.9 MPa to 73.1 MPa in the unreinforced mortar and from 110.6 MPa to 60.3 MPa in the polypropylene fibre-containing mortar. Up to temperatures of ca. 300 °C, the presence of PP fibres brought benefits in terms of both energy efficiency and mechanical properties, as it reduced stress concentration and delayed the initiation of micro-damage. At higher temperatures, however, this effect was reversed, which can be attributed to the loss of fibre continuity due to melting, accompanied by an increase in porosity and phase discontinuity within the cement matrix.

The modulus of elasticity tests showed that up to approximately 200 °C, fibre-free mortars were characterised by a more compact structure and lower deformability, indicating the dominance of chemically and physically bound hydration bonds within the cement matrix. Beyond this temperature range, fibre-reinforced mortars exhibited lower deformability, which may be ascribed to a change in the mechanism of strain energy transfer within the material, associated with the formation of channels after fibre melting and partial reorganisation of the silicate structure of the C–S–H gel.

From the point of view of engineering application, for structural elements primarily subjected to tensile loading, such as hollow-core slabs, the use of polypropylene fibres can be considered justified. Their presence improves both the mechanical properties at ambient temperature and the material behaviour under fire exposure by promoting a favourable redistribution of fracture energy.

A different situation may be observed in the case of elements primarily subjected to compressive loading, such as masonry blocks. Although PP fibres increase compressive strength at ambient temperature, at elevated temperatures their presence leads to a decline in load-bearing capacity of the composite. This results from the energetically unfavourable loss of structural continuity and an increased proportion of voids formed after fibre degradation.

Beyond the experimental aspect, the conducted research provides a significant contribution to the understanding of chemical and energy-related mechanisms occurring in cementitious composites containing polypropylene fibres under thermal exposure. The combined mechanical and microstructural investigations have provided crucial insight into the mechanisms controlling the behaviour of high-performance mortars, highlighting the influence of fibre degradation, phase discontinuities, and pore formation on their mechanical response. In particular, proof has been found that the presence of PP fibres reduces microcrack formation within concrete pores under fire conditions by creating microchannels that allow water vapour to escape and thereby reduce pore pressure.

SEM observations revealed no signs of deterioration within the fibre–cement paste interfacial zone. The molten polymer, driven by surface energy gradients and capillary phenomena, penetrates the surrounding porous cement matrix. As a result, in the temperature range above 300 °C, the role of polypropylene fibres undergoes a complete transformation: from active dispersed reinforcement at low and moderate temperatures, to a structural component whose melting and migration lead to a beneficial reorganisation of the cement composite microstructure under high-temperature conditions.

Moreover, it was also demonstrated that the beneficial effect of polypropylene fibres is limited to a moderate temperature range, up to approximately 300 °C. Above this threshold, chemical processes of dehydration of cement hydration products, degradation of the C–S–H gel, and unfavourable microstructural reorganisation become dominant, leading to a reduction in mechanical strength, particularly in compression. This limitation should be considered in the design process and indicates the need for further optimisation of fibre type and thermal stability to ensure reliable performance in engineering applications.

Overall, the findings of this study follow the trends commonly reported for cement-based mortars and concretes exposed to elevated temperatures. The results demonstrate the characteristic initial improvement in strength at relatively low temperatures (approximately 200 °C), generally associated with further hydration processes and moisture redistribution, followed by progressive deterioration of mechanical properties at higher temperatures. The deviations from the general trend observed at 300 °C may be linked to temporary structural stiffening caused by water evaporation and microscale rearrangement of the cement paste. Overall, the results fit well within the well-documented mechanisms describing the behaviour of PP fibre-reinforced cementitious composites under thermal exposure, while also extending the existing knowledge by incorporating energetic and microstructural aspects.

## Figures and Tables

**Table 1:** Physicochemical characteristics of CEM I 42.5R cement based on the manufacturer’s technical data sheet [59].

Property	Unit	Average Result	Requirements
Start of binding	Min	233	>60
End of binding	Min	291	
Water demand	%	27.5	
Volume stability	Mm	1.1	<10
Specific surface area	cm^2^/g	3688	
Compressive strength: after 2 days	MPa	23.9	<10
Compressive strength: after 28 days	MPa	55.9	>42.5, <62.5
Chemical analysis: SO_3_	%	2.77	<3.0
Chemical analysis: Cl	%	0.070	<0.10
Chemical analysis: Na_2_O eq.	%	0.53	<0.6

**Table 2:** Product data sheet specifications for the basic properties of microsilica [60,61,62,63].

Parameter	Unit	Value	Evaluation Method
Form	-	Fine-grained powder	Visual
Colour	-	Grey	Visual
Odour	-	Odourless	-
Density	g/cm^3^	2.05	PN-EN 1097-6 [61]
Bulk density	g/cm^3^	1.1	PN-EN 1097-3 [62]
Alkalinity	pH	Less than 11.5	PN-EN-ISO 10523 [63]

**Table 3:** Physicochemical properties of Chrysofluid Optima 185 according to the technical data sheet [56].

Feature	Description
Form	Liquid
Colour	Light brown
Density	1070 ± 20 kg/m^3^
pH	6.5 ± 1
Contents of Cl^−^	≤0.1%
Contents of Na_2_O	≤1.5%
Raw material base	Polycarboxylic ethers

**Table 4:** Properties of Ignis and Fibrofor High Grade fibres deployed in the testing [56].

Property	Fibre Name	
	Ignis	Fibrofor High Grade 190
Colour	Transparent	Beige
Characteristic	Monofilament	Bound, fibrillated
Length, [mm]	12	19
Film thickness, [μm]	18	80
Density, [g/cm^3^]	0.91	0.91
Tensile strength, [N/mm^2^]	min 28 cN tex^−1 a^	~400
Softening temperature, [°C]	~165	~150

^a^ cN tex^−1^—tensile strength unit for fibre, where tex is a unit for the linear mass density of fibres.

**Table 5:** Cement mortar compositions [56].

Components	Abbreviation		
	0F	1.8F	1.8I
Cement CEM I 42.5 R, [kg/m^3^]	846	846	846
Silica, [kg/m^3^]	84.6	84.6	84.6
Sand, [kg/m^3^]	1249	1249	1249
Optima 185 plasticiser, [%]cement mass	2	2	2
Water, [dm^3^]	215	215	215
Polypropylene fibres, [kg/m^3^]	0	1.8	1.8

**Table 6:** Compressive strength test results after 28 days of curing.

Sample Identification	Sample Dimensions [mm]	Weight [kg]	Density [kg/m^3^]	Compressed Area [mm^2^]	Failure Load [kN]	Compressive Strength [MPa]	Average Value [MPa]
Z0F1	99.8 × 100.8 × 100	2.250	2237	10,060	863.2	85.8	82.2
Z0F2	99.8 × 101.7 × 100.3	2.252	2208	10,145	848.6	83.6	
Z0F3	100 × 101 × 100.9	2.261	2216	10,102	781.2	77.3	
Z1.8F1	100 × 100.5 × 99.9	2.228	2219	10,050	815.9	81.2	79.9
Z1.8F2	100.2 × 100.1 × 100.2	2.201	2190	10,030	771.4	76.9	
Z1.8F3	99.7 × 99.8 × 100	2.221	2232	9950	813.4	81.7	

**Table 7:** Compressive strength test results after 90 days of curing.

Sample Identification	Sample Dimensions [mm]	Weight [kg]	Density [kg/m^3^]	Compressed Area [mm^2^]	Failure Load [kN]	Compressive Strength [MPa]	Average Value [MPa]
Z0F1	100.1 × 100.3 × 100	2.254	2245	10,040	935.7	93.2	94
Z0F2	100.2 × 100.7 × 99.8	2.211	2196	10,090	983.8	97.5	
Z0F3	101.1 × 100 × 100.2	2.230	2201	10,110	923	91.3	
Z1.8F1	100.5 × 100.2 × 99.9	2.234	2221	10,070	857	85.1	89.1
Z1.8F2	99.9 × 100.1 × 100	2.228	2228	10,000	935	93.5	
Z1.8F3	99.8 × 100.2 × 100.1	2.232	2230	10,000	887	88.7	

**Table 8:** Comparative analysis of compressive strength after 28 and 90 days of conditioning depending on specimen type: reference and PP fibre-reinforced specimens. Only for specimens conditioned for 90 days can it be stated at a statistically significant level of (1 − α)·100% = 90% that fibre-reinforced specimens exhibit lower compressive strength than the reference specimens. For 28-day conditioning, the inference is uncertain, at a level of 75%. For comparative purposes, an additional right-tailed *t*-test was performed, which means that the alternative hypothesis reads: the mean value of the first group is greater than the second group to compare.

Compressive Strength [MPa]	Shapiro Test (*p*-Value)	Fisher Test (*p*-Value)	Comparative *t* Test (*p*-Value)
0F after 28 days	0.48	0.527	0.24
1.8F after 28 days	0.58		
0F after 90 days	0.18	0.725	0.09
1.8F after 90 days	0.84		

**Table 9:** Results of a statistical comparison of flexural tensile strength for mortar specimens reinforced with 1.8 kg/m^3^ of F fibres and reference specimens at different temperatures. At an acceptable significance level (above 90%), it can be concluded that only at 20 °C and 100 °C are fibre-reinforced specimens more resistant than reference specimens. At higher temperatures, the inference is uncertain, although the mean values are indeed higher. A one-sided Shapiro–Wilk test was performed to verify whether the distribution of values within the sample can be considered Gaussian, as well as tests comparing means at each temperature level. The alternative hypothesis stated that: “the mean tensile strength of reference samples is lower than that of F-fibre-reinforced samples.”.

Tensile Strength for Samples 0F or 1.8F	Mean ± Sd	Shapiro Test (*p*-Value)	Fisher Test (*p*-Value)	Comparative *t* Test (*p*-Value)
0F at 20 °C	8.4 ± 1.2	0.663	0.188	0.004
1.8F at 20 °C	10.7 ± 0.6	0.468	0.468	
0F at 100 °C	9.4 ± 1.2	0.936	0.046	0.056 ^(^*^)^
1.8F at 100 °C	12.6 ± 3.5	0.136	0.136	
0F at 200 °C	7.7 ± 0.9	0.408	0.172	0.232
1.8F at 200 °C	8.5 ± 1.8	0.135	0.135	
0F at 300 °C	8.8 ± 0.8	0.229	0.183	0.463
1.8F at 300 °C	8.9 ± 1.7	0.118	0.118	
0F at 400 °C	6.7 ± 0.7	0.265	0.542	0.400
1.8F at 400 °C	6.8 ± 1.0	0.541	0.541	
0F at 500 °C	6.3 ± 0.6	0.286	0.110	0.607 ^(^**^)^
1.8F at 500 °C	6.9 ± 1.6	0.071	0.071	
0F at 600 °C	5.1 ± 0.4	0.530	0.017	0.747 ^(^*^)^
1.8F at 600 °C	4.6 ± 1.6	0.991	0.991	

(*) As the variances were statistically significantly different, Welch’s *t*-test was applied instead of the standard *t*-test; (**) due to uncertainty regarding the normality of the distribution for mortar 1.8F at 500 °C (*p*-value only marginally above the adopted significance level α), the Wilcoxon test was performed.

**Table 10:** Results of the statistical comparison of the coefficient of variation for mortars without fibre addition, 0F, and mortars with fibre addition, 1.8F, and correlation analysis of CV vs. temperature. Columns 2 and 3 are the results of the preliminary analysis necessary to correctly conduct the comparative analysis (selection of the statistical test, column 4). Column 5 presents the results of the one-sided test for the correlation coefficient of CV vs. temperature. The null hypothesis was excluded for the alternative hypothesis of a negative correlation whenever the *p*-value was below the adopted significance level (α). A 95% confidence interval for the correlation coefficient was also calculated. The outcome of the corresponding two-sided test is reported in the final column.

	Shapiro Test	Fisher Test	Comparison Test	Correlation CV vs. T *p*-Value; 95% Conf. Int.	Correlation CV vs. T *p*-Value; Two-Sided
CV for F at 0.0 kg/m^3^	0.98	0.001	0.015	0.002; [−0.98, 0.52]	0.004
CV for F at 1.8 kg/m^3^	0.99			0.90; [−0.34, 0.92]	0.19

**Table 11:** Results of linear regression for compressive strength as a function of temperature for mortars without fibre addition and with polypropylene (PP) fibre addition.

Quantity	Fibre Density	Slope ± SE	Intercept ± SE	R Squared
Compressive After bending	0.0 kg/m^3^	−0.051 ± 0.005	105 ± 2	0.65
	1.8 kg/m^3^	−0.092 ± 0.006	115 ± 2	0.79

**Table 12:** Statistical comparison of the coefficient of variation between mortar groups without fibre addition and with polypropylene (PP) fibre addition for compressive strength, along with the estimated correlation coefficient and its 95% confidence interval for CV versus temperature.

	Shapiro Test	Fisher Test	Comparison Test	Correlation CV vs. T *p*-Value; 95% Conf. Int.
CV for F at 0.0 kg/m^3^	0.07	0.836	0.18	0.84; [−0.79, 0.71]
CV for F at 1.8 kg/m^3^	0.09			0.62; [−0.84, 0.63]

**Table 13:** Statistical analysis of the coefficient of variation for mortars without PP fibre addition and for mortars reinforced with F fibres at a dosage of 1.8 kg/m^3^.

Quantity	Fibre Density	Shapiro Test, *p*-Value	Correlation Coefficient	Correlation Test, *p*-Value	95% Conf. Int. for Correlation	Fisher Test, *p*-Value	*t* Test, *p*-Value
Coefficient of variation for the modulus of elasticity	0.0 kg/m^3^	0.75	−0.311	0.75	(−0.86, 0.58)	≈1	0.43
	1.8 kg/m^3^	0.11	0.846	0.008	(0.404, 1.00)	≈1	

## Data Availability

The raw data supporting the conclusions of this article will be made available by the authors on request.
